# Quantifying the Effect Size of Management Actions on Aboveground Carbon Stocks in Forest Plantations

**DOI:** 10.1007/s40725-023-00182-5

**Published:** 2023-04-11

**Authors:** Cyril H. Melikov, Jacob J. Bukoski, Susan C. Cook-Patton, Hongyi Ban, Jessica L. Chen, Matthew D. Potts

**Affiliations:** 1 Environmental Defense Fund, New York, NY USA; 2 Department of Environmental Science, Policy and Management, University of California, Berkeley, CA USA; 3 Moore Center for Science, Conservation International, Arlington, VA USA; 4 Department of Forest Ecosystems and Society, Oregon State University, Corvallis, OR USA; 5 The Nature Conservancy, Arlington, VA USA; 6 Carbon Direct Inc, New York, NY USA

**Keywords:** Natural climate solutions, Improved forest management, Carbon, Fertilization, Thinning, Forest plantation

## Abstract

**Purpose of the Review:**

Improved forest management is a promising avenue for climate change mitigation. However, we lack synthetic understanding of how different management actions impact aboveground carbon stocks, particularly at scales relevant for designing and implementing forest-based climate solutions. Here, we quantitatively assess and review the impacts of three common practices—application of inorganic NPK fertilizer, interplanting with N-fixing species, and thinning—on aboveground carbon stocks in plantation forests.

**Recent Findings:**

Site-level empirical studies show both positive and negative effects of inorganic fertilization, interplanting, and thinning on aboveground carbon stocks in plantation forests. Recent findings and the results of our analysis suggest that these effects are heavily moderated by factors such as species selection, precipitation, time since practice, soil moisture regime, and previous land use. Interplanting of N-fixing crops initially has no effect on carbon storage in main tree crops, but the effect becomes positive in older stands. Conversely, the application of NPK fertilizers increases aboveground carbon stocks, though the effect lessens with time. Moreover, increases in aboveground carbon stocks may be partially or completely offset by emissions from the application of inorganic fertilizer. Thinning results in a strong reduction of aboveground carbon stocks, though the effect lessens with time.

**Summary:**

Management practices tend to have strong directional effects on aboveground carbon stocks in plantation forests but are moderated by site-specific management, climatic, and edaphic factors. The effect sizes quantified in our meta-analysis can serve as benchmarks for the design and scoping of improved forest management projects as forest-based climate solutions. Overall, management actions can enhance the climate mitigation potential of plantation forests, if performed with sufficient attention to the nuances of local conditions.

**Supplementary Information:**

The online version contains supplementary material available at 10.1007/s40725-023-00182-5.

## Introduction

Mitigating the most damaging effects of climate change requires concerted and urgent action within this decade in both the energy and land sectors [1]. Recently, studies have highlighted the potential of tree cover restoration for mitigating climate change, in places where formerly forested landscapes have been lost or severely degraded [2–6]. Despite momentum to restore tree cover, improving the management of existing forests may represent a more cost-effective and rapidly deployable natural climate solution, and could sequester 0.1 to 2.3 PgCO_2_e per year [1, 5, 7•, 9••]. Given limited resources and the urgency of climate change, identifying near-term management actions that can maximize carbon stocks in managed forest stands is of paramount importance.

In natural forests, multiple studies have focused on how improved forest management practices, such as reduced-impact logging and liana control, can increase aboveground carbon stocks, relative to standard forestry practices [5, 8•, 10–12]. In planted forests, studies suggest that extending rotations to a biological rather than economical optimum can substantially increase time-averaged carbon stocks [5, 12]. Nonetheless, forestry practitioners commonly perform additional operations in planted stands such as fertilization and stand density management that directly influence plantation carbon stocks [13••, 15••]. Here, we consider the effects of two fertilization practice-intercropping of nitrogen (N)-fixing species and application of inorganic nitrogen, phosphorus, potassium (NPK) fertilizer-and one stand density management operation (thinning) on aboveground carbon stocks in forest plantations.

While the use of NPK fertilizers remains the most common fertilization practice [15••], interplanting of N-fixing plants as an alternative method has become increasingly prominent in mixed species plantations [16, 17, 18•, 19]. In addition to providing N-fixing benefits, planting of multiple species may confer additional ecological benefits, such as habitat for biodiversity and improved soil fertility [15••, 18•, 20, 21]. Furthermore, the use of conventional inorganic NPK fertilizers has potentially adverse environmental impacts from improper and over-use, including declines in soil fertility, elevated groundwater and surface water pollution, and increased GHG emissions from fertilizer production and use [22, 23].

The empirical effects of these fertilization techniques on aboveground carbon stocks in plantations has been well documented in multiple locations [24–29]. However, the magnitude of the effect has been rarely assessed at large scales [17, 30, 31] relevant for the design of forest-based climate solutions, with prior efforts constrained to regional geographies [32] or specific tree species [16]. Furthermore, these prior large-scale meta-analyses are now more than 10 years old, and to the best of our knowledge, none has examined moderators of the fertilizing effect nor investigated how the effect size might vary with site and stand characteristics.

Similarly, the effects of thinning stand biodiversity [33, 34], soil carbon stocks [35, 36], soil microbial carbon [37], and even drought-related tree stress [38] have been studied at site-level scales. However, only a handful of studies have synthesized the changes in plantation aboveground carbon stocks after thinning operations in large-scale meta-analyses [39–41], and most of the previous investigations were plot-scale empirical studies [42–44]. Similar to fertilization treatments, few studies have investigated how thinning effects change with site characteristics, with most of the past efforts focusing on the impacts of stand age and time since treatment on the treatment’s effect size [39, 40].

The global effects of these silvicultural treatments as well as the contexts under which they deliver the most carbon benefits still need to be documented. Our work builds on these previous empirical studies and meta-analyses. It aims to improve understanding of how prominent silvicultural practices impact aboveground carbon stocks in plantations globally. To do so, we systematically reviewed and compiled aboveground carbon measurements from interplanted, fertilized, and thinned tree plantations distributed across six continents and 18 countries. Using this dataset, we then quantified (i) how each treatment affected aboveground carbon stocks and (ii) how the effect size of each treatment varied with different environmental and management factors. In doing so, we provide insights on how fertilization and thinning treatments can be improved to promote carbon stocks in planted forests.

## Methods

### Literature Search and Data Collection

**Fig. 1:**
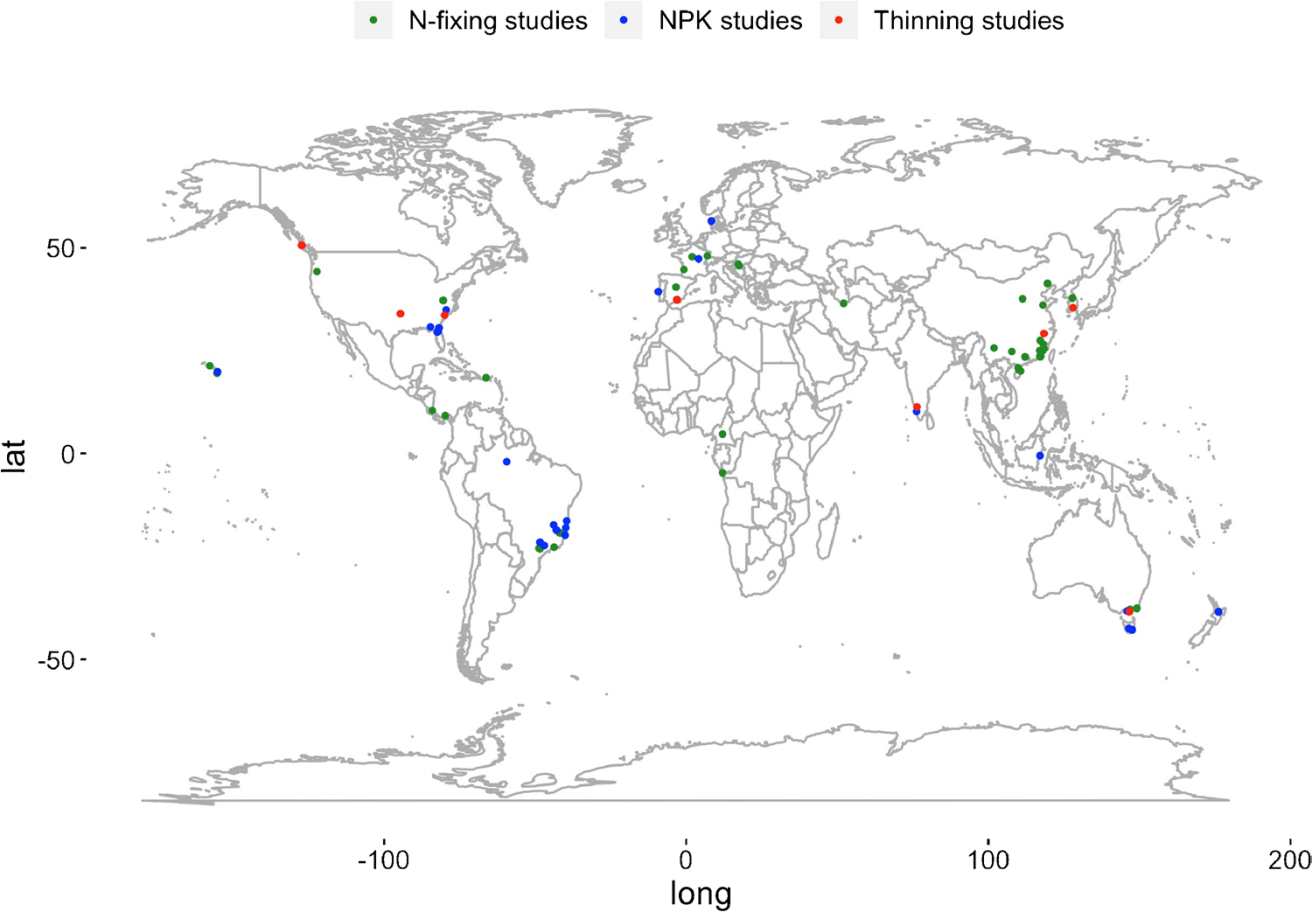
Map of locations of all sites included in the meta-analysis of intercropping N-fixing plants, NPK fertilization, and thinning effects on planted forests aboveground carbon stocks

We conducted this meta-analysis using a recently published global database that compiles 4756 measurements of aboveground live tree carbon stocks in timber plantations, collected from 829 distinct sites across 278 studies [45••]. We subset this dataset to the 654 measurements of aboveground live tree biomass in timber plantations across 45 studies, 56 distinct sites, and 19 tree genera that included relevant management details. Full details on the monoculture plantation database compilation and data standardization processes are described in Bukoski et al. [45••]. In addition, we included 97 measurements of aboveground live trees biomass collected from one large compilation of aboveground carbon stocks in planted forests [46••] that was found at a later date. We elected to include it as it substantially increased our sample size. In total, we collected 751 measurements of aboveground live tree biomass across 68 studies (Table S1.1, S1.2, and S1.3), 80 distinct sites, and 19 tree genera (Fig. 1).

For each of the three dominant silvicultural treatments—intercropping of N-fixing species, application of inorganic NPK fertilizer, and stand density management (i.e., thinning), we selected studies from our monoculture plantation database that had both (i) measurements of aboveground biomass from plots in which the treatment had been applied and (ii) measurements of aboveground biomass from control plots (without the treatment of interest). Only studies with imposed, replicated treatments at one or more sites were included in our dataset. Multiple comparisons within a single study (e.g., comparing different thinning intensities to a single unthinned control) were considered as distinct within-study observations, and in that case, treatment values were compared to the same control value. In total, there were 43 studies comprising 197 comparisons for intercropping of N-fixing plants, 17 studies comprising 164 comparisons for NPK fertilization, and 8 studies comprising 62 comparisons for thinning (the included studies are in Tables S1.1 & Table S1.2 & Table S1.3). Within the intercropped plots, we conservatively accounted for aboveground biomass of only the main tree crops to assess the fertilization effect of the interplanted N-fixing plants on the latter. Before running the analysis, we converted aboveground live tree biomass measurements to aboveground carbon using a 0.47 default conversion factor [47].

### Standardization of Treatment Effect Using Natural Log of the Response Ratio

For our meta-analysis, we used the natural log of the response ratio metric (lnRR) to standardize the effect of treatments on aboveground carbon across studies. Here, *lnRR* reflects the change in aboveground carbon induced by each of our three treatments—interplanting of N-fixing species, application of NPK fertilizers, and thinning. We calculated *lnRR* using Eq. (1):

1 $$lnRR=ln\frac{\overline{Xt} }{\overline{Xc} }=ln\overline{Xt }-ln\overline{Xc }$$

In Eq. (1), $\overline{Xt }$ and $\overline{Xc }$ are mean aboveground carbon in the paired treatment and control, respectively. A *lnRR* value of 0 means the treatment did not induce any change in carbon compared to the control, while a positive value indicates the treatment had a positive effect on aboveground carbon, and a negative value indicates a decrease in aboveground carbon. To account for variation in sampling effort between studies, we weighted the effect sizes by the inverse of the sample variance for each response ratio. We calculated the sample variance using the standard error and number of replicates reported for each study [48]. When studies did not report the standard deviations associated with aboveground carbon measurements, we followed the methodology of Lajeunesse [49] and Koricheva [50]. This consisted of imputing the missing standard deviations by calculating the median coefficient of variation (ratio of standard deviation and mean) for each group (treatment or control) from studies that reported both means and standard deviations. We then multiplied the median coefficient of variation by the reported mean for either treatment or control groups for which standard deviations were missing. We performed a sensitivity analysis to test for any potential effects of these assumptions on our results (see Supplementary Information), which revealed that almost all results were robust to this approach, but we note where results were sensitive to the imputed standard deviations. Finally, we used funnel plots to confirm the absence of publication bias [51]. Response ratios were calculated using the {metaphor} package [52] in Program R v.1.4.1103 [53].

### Testing of Moderator Effects and Mixed-Effects Approach

We inserted several moderators (variables that influence the strength and/or form of a relationship between a predictor and a dependent variable [54]) into the mixed-effects model to assess how the treatment effect size varied with other factors hypothesized to influence aboveground carbon (e.g., genus or soil moisture regime). Our selection of moderator variables included both categorical and continuous moderators and sought to account for an array of environmental, biological, and human factors, but was also limited by data availability (Table 1). Except soil moisture regime, all moderator variables were recorded using information reported in the studies themselves. To obtain soil moisture regimes, we intersected the geographic coordinates of each site with a map of global soil moisture regimes developed by USDA-NRCS [55]. This map of global soil moisture regimes was built using data taken from more than 22,000 climatic stations distributed around the world [55]. Soil moisture regime data were interpolated and rasterized on a 2-min grid cell [55].

**Table 1:** Moderator variables inserted in the mixed-effects meta-analytic models for each treatment

Treatments	Moderator	Moderator type	Categories/units
Interplanting N-fixing plants	Previous land use	Categorical	Cropland, natural forest, plantation
	Tree genus	Categorical	*Alnus*, *Anacardium*, *Casuarina*, *Eucalyptus*, *Hymeronima*, *Pachira*, *Pinus*, *Populus*, *Pseudotsuga*
	Soil moisture regime	Categorical	Perudic, udic, ustic, xeric
	Intercropped plant genus	Categorical	*Acacia*, *Albizia*, *Alnus*, *Dalbergia*, *Enterolobium*, *Hippophae*, *Leucaena*, *Lupinus*, *Paraserianthes*, *Robinia*, *Salix*
	Wood type	Categorical	Hardwood, softwood
	Experimental design	Categorical	Additive, replacement
	Stand age	Continuous	Years
	Mean annual precipitation	Continuous	mm/year
Inorganic NPK fertilization	Previous land use	Categorical	Cropland, fire, natural forest, plantation
	Tree genus	Categorical	*Ailanthus*, *Eucalyptus, Macaranga*, *Picea*, *Pinus*
	Soil moisture regime	Categorical	Perudic, udic, ustic, xeric
	Wood Type	Categorical	Hardwood, softwood
	Stand age	Continuous	Years
	Time since fertilization Application method	Continuous Categorical	Years Continuous, pulse
	Mean annual precipitation	Continuous	mm/year
Thinning	Previous land use	Categorical	Cropland, natural forest, plantation
	Soil moisture regime	Categorical	Perudic, udic, ustic, xeric
	Wood Type	Categorical	Hardwood, softwood
	Tree genus	Categorical	*Acacia*, *Cunninghamia*, *Eucalyptus*, *Pinus*
	Time since thinning	Continuous	Years
	Mean annual precipitation	Continuous	mm/year
	Basal area removed	Continuous	Percent

Moderators were tested individually with separate models, such that the influence of a moderator on the effect size was determined using all available data for that particular moderator variable. Prior to analysis, we dropped categories of moderators for which we had only one observation. To account for the non-independence of multiple within-study observations, we inserted publication-level random effects into each of our models [50]. We fitted all models using restricted maximum likelihood estimation [50]. We determined the statistical significance of each moderator variable using an omnibus test of all model coefficients (*p*-value < 0.05) [52]. Prior to reporting our final results, we back-transformed (e^lnRR^) the mean log response ratios and their 95% confidence intervals and converted these values to percent change relative to the control.

Depending on data availability, we also tested how the effect size varied with either tree age or time since treatment using linear mixed-effects models. In all the interplanting treatment studies, N-fixing crops were planted at the same time as the main tree crop, and we therefore used stand age as our time variable. To limit the potential effects of sparse data at older ages, we dropped measurements above 20 years old before performing this regression. In total, 2 data points at 21 and 28 years old were dropped prior to this linear regression analysis. Of the inorganic fertilizer studies, 9 of 18 continuously applied inorganic fertilizer over the course of the study period and we used stand age as our time variable. However, 10 of 18 inorganic fertilizer treatment studies reported “time since treatment” data. For these observations, we also used mixed-effects regression models to test how the effect size varied with time since treatment in inorganically fertilized plots (Table 1). Limited data were available for older times since NPK fertilization. To limit the potential effects of sparse data at older times since treatment, we dropped measurements above a time since treatment of 5 years before running this regression. In total, two data points at 21 years old were dropped prior to this linear regression analysis. For our thinning observations, we assessed how the effect size varied with time since treatment in thinned plots as well (Table 1). We used all the time since thinning data to perform the regression analysis. Finally, we evaluated whether annual mean precipitation had an influence on the effect size of all three treatments using the same regression model type (Table 1).

### Greenhouse Gas Emissions from Inorganic Fertilizer Application

For each aboveground carbon measurement reported in NPK fertilization studies, we calculated the business as usual (BAU) associated amount of nitrous oxide (N_2_O) emitted (expressed in CO_2_e) for each ton of synthetic nitrogen (N) fertilizer applied, both in-field and upstream from fertilizer manufacturing itself [5]. We used an emissions factor of 2.54% for N fertilizer (11.9 MgCO_2_e per MgN applied) for in-field emissions and an upstream emissions factor of about 4 kgCO_2_e per kgN produced [5]. We extracted tonnage of N fertilizer directly from the studies included in this meta-analysis (Table S1.2). When the amount of N fertilizer used was reported as kilograms per tree, we converted to kilograms per hectare using the reported stand density value. We then compared the additional aboveground carbon gain induced by NPK fertilization (i.e., the difference in aboveground carbon between the treated and control plots) with emissions from N fertilizers use and manufacturing. We calculated the difference between additional carbon and N fertilizer emissions and examined how this effect varied with the amount of N fertilizer applied using a linear regression model. Finally, we estimated the median net carbon balance of fertilized stands (expressed MgCO_2_e ha^−1^) across all studies included for the NPK fertilization treatment.

## Results

Our results showed effects on aboveground carbon that were (i) strongly positive and statistically significant for N-fixing species, (ii) strongly positive and statistically significant for the use of NPK fertilizer, and (iii) negative and statistically significant for thinning. Further, our incorporation of moderator variables provided additional nuance on how these silvicultural practices impact aboveground carbon in plantations. We provide additional details on each of the three treatments below.

### Interplanting of N-Fixing Species

**Fig. 2:**
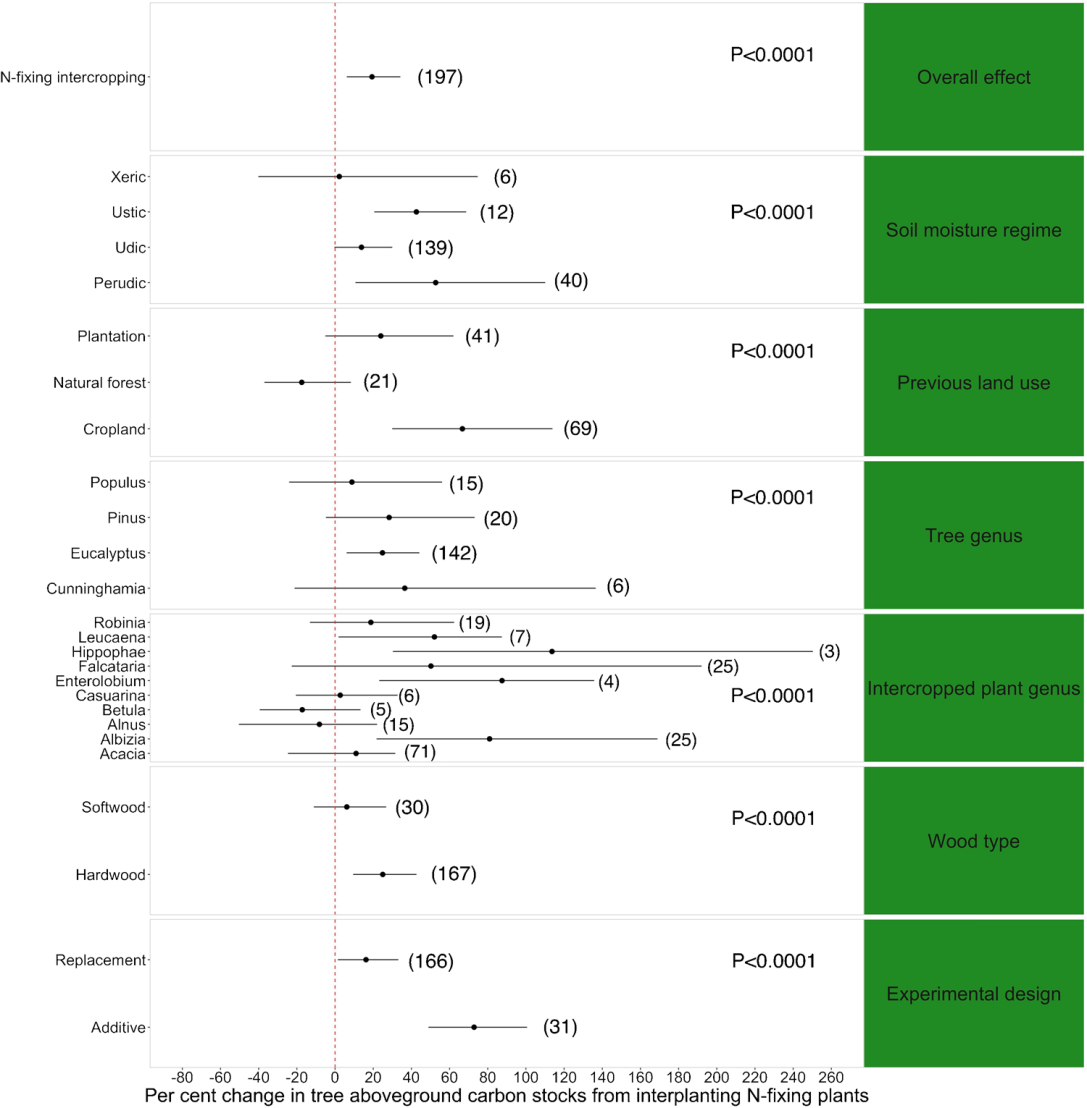
Meta-analysis results of the change in aboveground carbon of plantation trees in response to the interplanting of N-fixing plants. Error bars represent the 95% confidence intervals. Omnibus tests of significance for moderator variables are shown on the right side (NS means “not significant”). Results for the “Tree genus” moderator are only provided for genera with more than 5 observations (see Table S5.1). Results for the “Intercropped Genus” moderator are only provided for genera with significant effects or for genera with more than 5 observations (see Table S5.1). The number of observations in each category is shown in parenthesis

Overall, intercropping of N-fixing plants in monoculture stands had a significantly positive effect (approximately + 20%) on aboveground carbon of the primary species (Fig. 2) (*p*-value = 0.0031). All moderator variables were found to have significant effects on the relationship between interplanted N-fixing crops and aboveground carbon (Fig. 2). We found that “soil moisture regime” had a strong influence on the magnitude of the effect size (*p* < 0.001) (Fig. 2). Interplanting of plantations growing on moister soils significantly increased aboveground carbon (+ 52% for perudic and + 42% for ustic soils), but not in places with drier xeric soil moisture regimes (Table S5.1). Furthermore, N-fixing companion crops significantly increased the aboveground carbon stocks of the main tree crop when plantations occurred on former croplands (+ 67%, *p* < 0.0001) (Fig. 2), rather than locations with tree cover. The genus of the main tree crop also influenced the magnitude of the effect size (*p* < 0.001). Specifically, establishing *Eucalyptus* trees with N-fixing companion crops increased the latter aboveground carbon stocks by + 25%, whereas other genera did not show a significant effect. Additionally, the genus of the intercropped species had a significant impact (*p*-value < 0.001) on the effect size as well (Fig. 2). Intercropping with *Leucaena*, *Albizia*, *Enterolobium*, and *Hippophae* induced a 52%, 80%, 87%, and 113% increase in the aboveground carbon of the main tree crop, respectively, whereas other intercropping genera did not have a significant effect (Fig. 2; Table S5.1). The type of wood of the main tree species also moderated the effect of the treatment (*p*-value = 0.0036). Hardwoods reacted positively to interplanted N-fixing crops (+ 25% in aboveground carbon stocks) (Table S5.1). Finally, the type of experimental design influenced the magnitude of effect size (*p*-value < 0.001) (Fig. 2). The use of interplanted N-fixing crops appeared to be more beneficial when an additive design was adopted (+ 72%) than a replacement one (+ 16%) (Table S5.1).

**Fig. 3:**
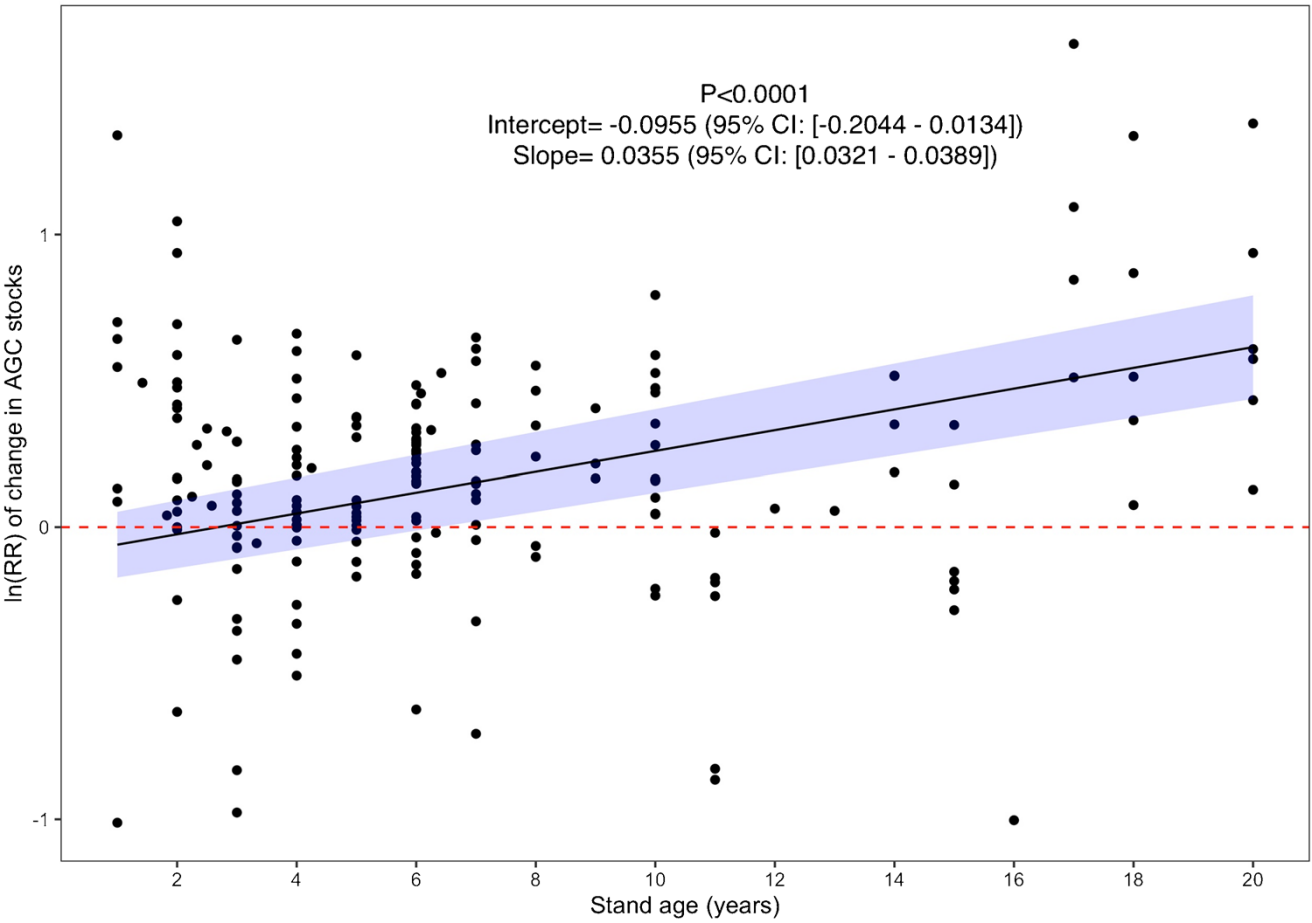
Change in the effect size of interplanting N-fixing plants as a function of stand age. The significance of the regression is indicated by the *p*-value in the upper right as well as the intercept and slope values with their corresponding 95% confidence interval. The area shaded in blue around the regression lines indicate the 95% confidence interval

However, our results also suggest that the effect of intercropping N-fixing species on aboveground carbon varied with stand age. By regressing ln(RR) on stand age, we identified a significant positive association with the treatment effect size (*p*-value < 0.0001) (Fig. 3). For every additional year that a stand was allowed to grow, the influence of intercropping N-fixing species on plantation aboveground carbon was increased by 3.6% (Fig. 3). Intercropping initially decreased aboveground carbon in the main tree crop, although not significantly, but its effect became positive when the stand was 3 years old and steadily increased over time from then on t (+ 84% at 20 years old) (Fig. 3).

Finally, we did not identify a significant effect of mean annual precipitation, the other continuous variable in our model, on the magnitude of the effect size (*p*-value = 0.0872; Table S5.1).

### Inorganic NPK Fertilization

**Fig. 4:**
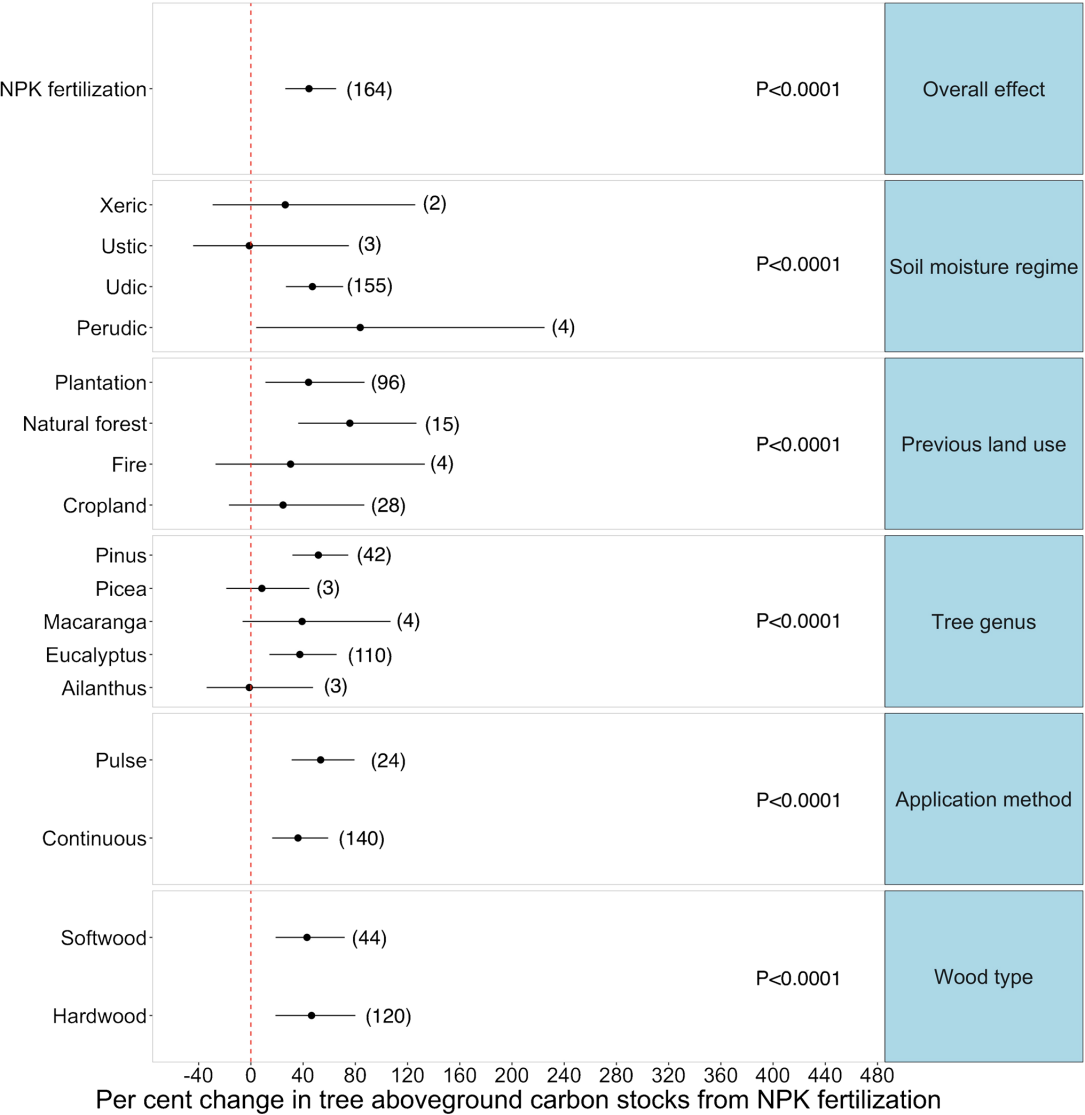
Meta-analysis results of the change in aboveground carbon of plantation trees in response to NPK fertilization error bars represent the 95% confidence intervals. Omnibus tests of significance for moderator variables are shown on the right side (NS means “not significant”). The number of observations in each category is shown in parenthesis

We found that inorganic NPK fertilizers significantly increased aboveground carbon by 44.5% overall (*p*-value < 0.001) (Fig. 4). Of the individual studies included in the meta-analysis, most reported a significant positive effect of NPK fertilization on aboveground carbon, whereas six studies found a non-significant effect. Similarly to interplanting of N-fixing species, all moderator variables were found to have significant effects on the relationship between NPK fertilizer and aboveground carbon.

We found that “tree genus” significantly influenced the change in aboveground carbon attributed to inorganic NPK fertilization (Fig. 4) (*p*-value < 0.001). The aboveground carbon of treated *Pinus* and *Eucalyptus* plantations were 51% (*p*-value < 0.001) and 37% (*p*-value < 0.001) higher in fertilized plots compared to the controls, respectively (Fig. 4; Table S5.2). In addition, both hardwoods and softwoods responded positively to the treatment; however, NPK fertilization had a slightly greater effect on aboveground carbon in hardwood (+ 46%) (*p*-value < 0.001) relative to softwood plantations (+ 43%) (*p*-value < 0.001) (Fig. 4; Table S5.2).

Soil moisture regime also had a strong influence on the magnitude of the effect size (*p*-value < 0.001) (Fig. 4). NPK fertilization of plantations growing on moister soils significantly increased aboveground carbon (+ 82% for perudic and + 42% for udic soils), but not in places with xeric and ustic soil moisture regimes (Table S5.2). It is worth noting that the influence on the effect size of the perudic soil moisture regime might be overestimated as only one study reported measurements for that soil moisture regime (Fig. 4). Furthermore, the methodology used to apply NPK fertilizers significantly influenced the magnitude of the fertilizing effect (*p*-value < 0.001) (Fig. 4). The effect size appeared higher when fertilizers are applied episodically (+ 53%) rather than continuously (+ 36%) (Table S5.2). The type of previous land use also strongly influenced the treatment effect size and explained part of the heterogeneity in effect size across studies (Fig. 4) (*p*-value < 0.001). Specifically, the use of inorganic fertilizers appeared to be more beneficial on lands that were previously plantations (+ 44%) or natural forests (+ 76%), than those cleared by fire or previously used for croplands (Table S5.2).

**Fig. 5:**
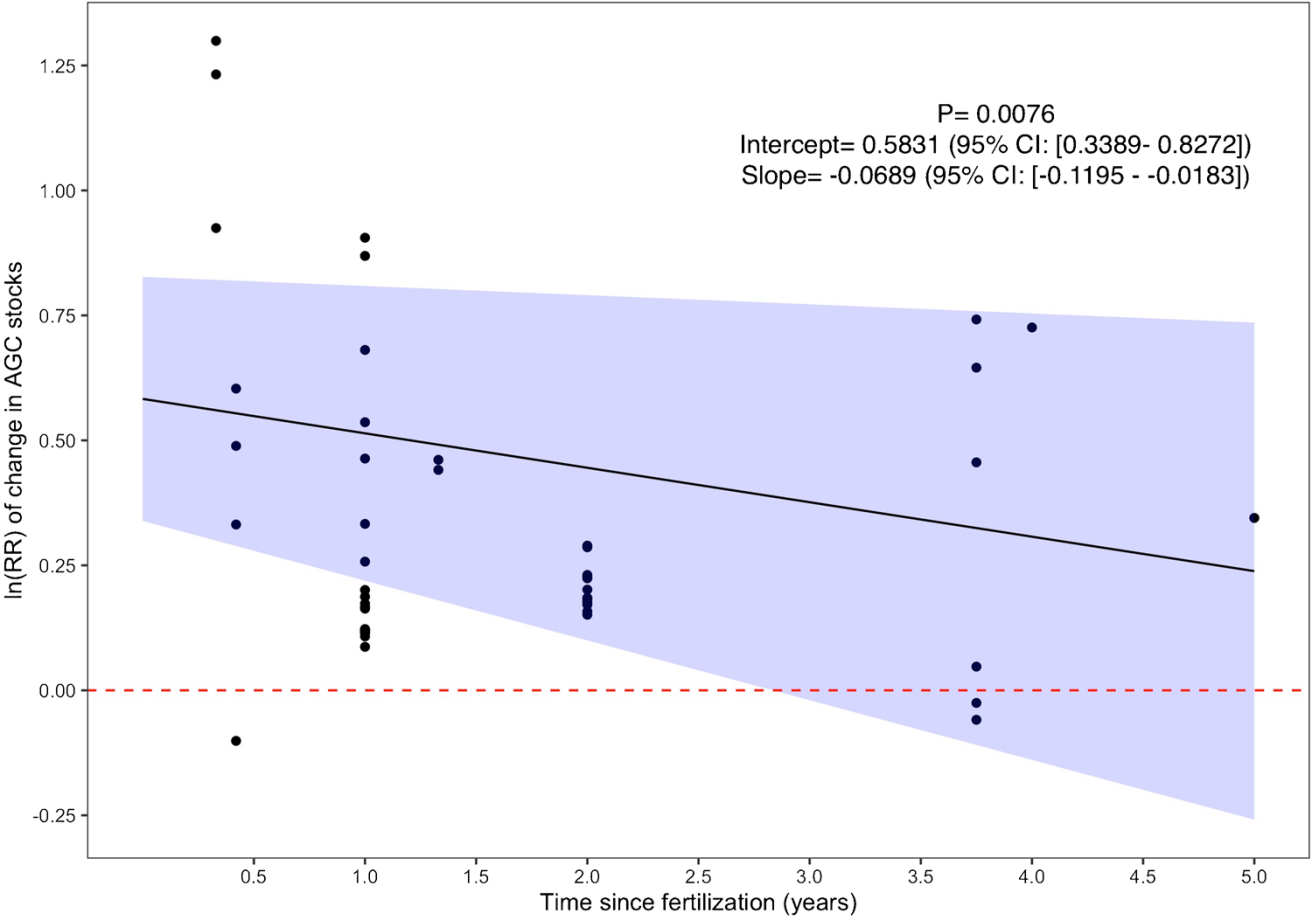
Change in the fertilizing effect size of NPK fertilizers as a function of time since treatment. The significance of the regression is indicated by the *p*-value in the upper right as well as the intercept and slope values with their corresponding 95% confidence interval. The area shaded in blue around the regression lines indicate the 95% confidence interval

We found a negative, although not significant (*p*-value = 0.5), association between stand age and the magnitude of the effect size in fertilized stands (Table S5.2). However, when we used time since treatment rather than stand age, we found that the benefit of inorganic fertilizers on aboveground carbon significantly decline through time (*p*-value = 0.008) (Fig. 5). For every additional year since the treatment, the effect size of inorganic fertilization on carbon stocks decreased by 6.66% (Fig. 5).

Mean annual precipitation also had a significant negative effect (*p*-value < 0.001) on the magnitude of the change in aboveground carbon (Figure S3.1). For every additional millimeter of precipitation received per year, the treatment effect size was reduced by 0.03% (Figure S3.1).

**Fig. 6:**
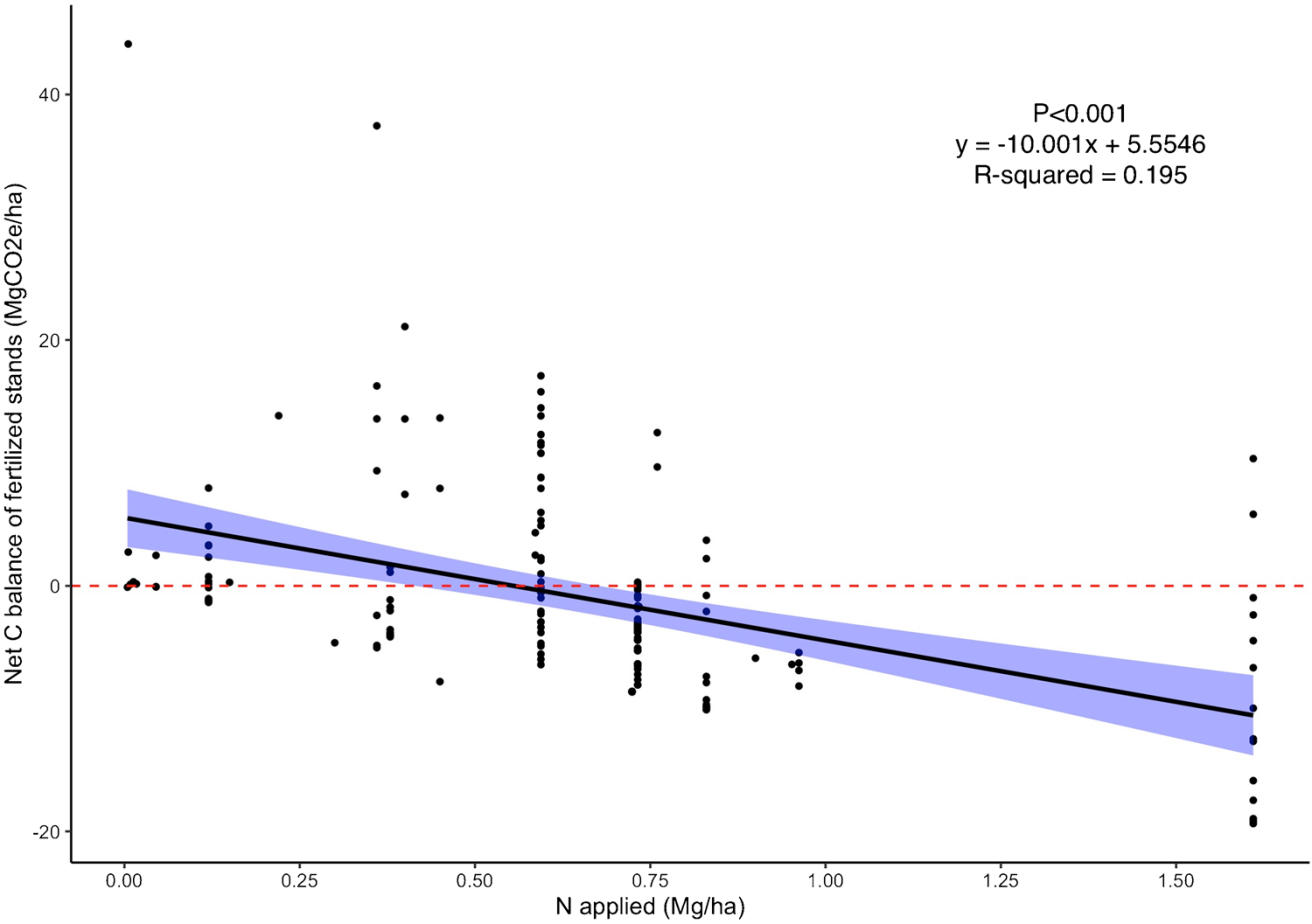
Change in the net carbon balance of fertilized stands as a function of the amount of N applied. The significance of the regression is indicated by the *p*-value in the upper right as well as the intercept and slope values, and the coefficient of determination. The area shaded in blue around the regression lines indicate the 95% confidence interval

Once we accounted for in-field and upstream fertilizer emissions, we found a negative median net carbon balance of fertilized stands across studies (− 2 MgCO_2_e.ha^−1^). In other words, fertilized stands were net emitters across studies (Figure S3.2), and the climate mitigation benefit of fertilizer declined with the amount of N fertilizer applied (*p*-value < 0.001). The climate benefit became null at 0.55 MgN or greater applied per hectare (Fig. 6).

### Thinning

**Fig. 7:**
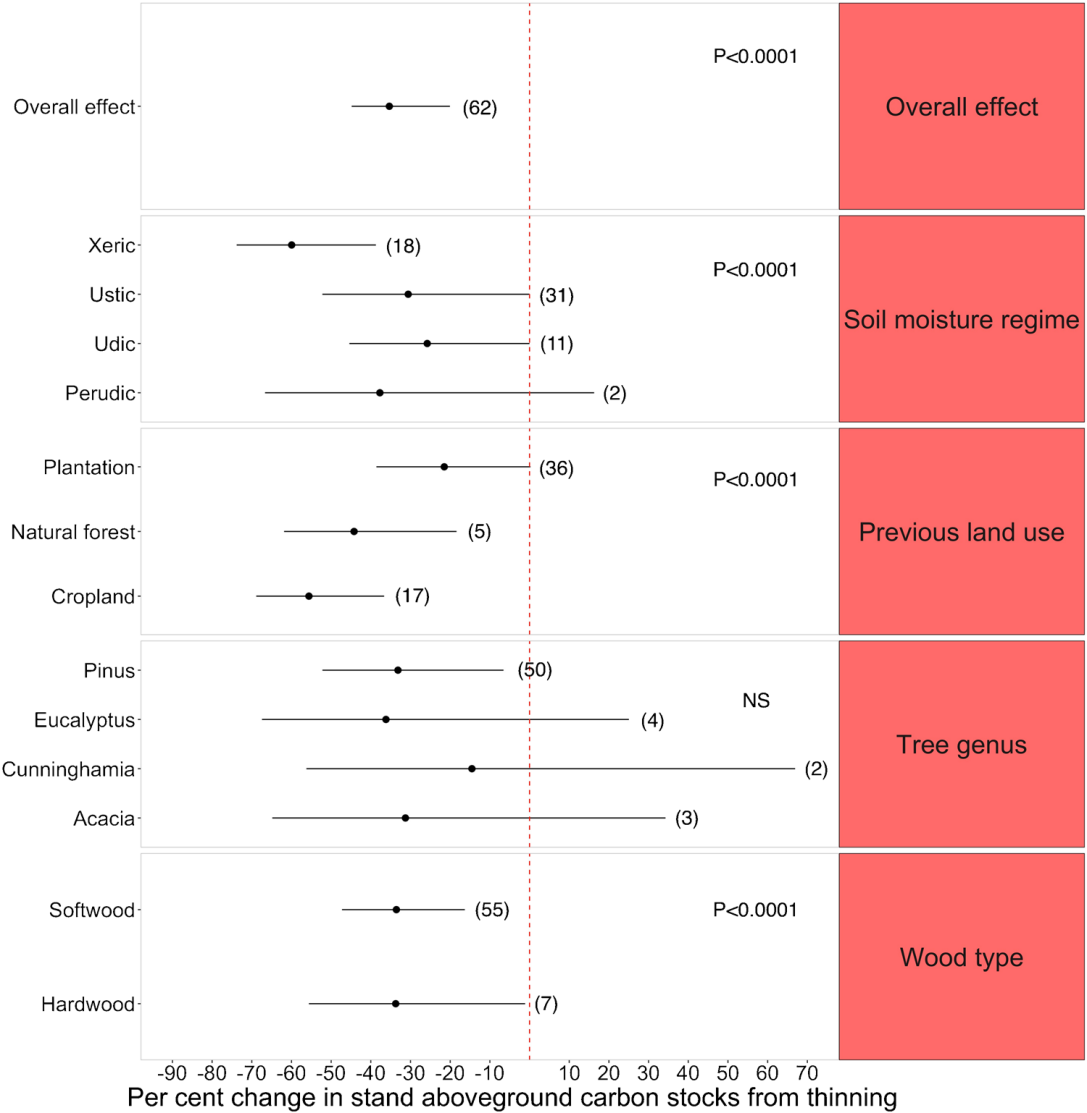
Meta-analysis results of the change in aboveground carbon of plantations from thinning. Error bars represent the 95% confidence intervals. Omnibus tests of significance for moderator variables are shown on the right side (NS means “not significant”). The number of observations in each category is shown in parenthesis

Across the different studies, thinning decreased standing aboveground carbon by about 34% (*p*-value < 0.001) (Fig. 7). We found soil moisture regime to have a significant impact on the percent change in carbon from thinning (*p*-value < 0.001) (Fig. 7). When thinning was conducted on xeric soils, carbon levels decreased significantly by 59% (*p*-value < 0.001) (Table S5.3)*.* We found type of previous land use to also have a significant influence on the magnitude of the effect size (*p*-value < 0.001) (Fig. 7). Stands grown on former agricultural lands had aboveground carbon levels that were 55% lower (*p*-value < 0.001) in thinned stands compared to controls (Table S5.3). When thinning was executed in stands that were established on former natural forestlands, the mean decrease in aboveground carbon was 44% (*p*-value = 0.0026) (Table S5.3). Finally, we also found that the type of wood influenced the magnitude of the effect size (*p*-value < 0.001) (Fig. 7). Both hardwood and softwood stands experienced large reductions in aboveground carbon due to thinning operations, which averaged a − 33% change in aboveground carbon from thinning for both wood types. Genus of tree did not significantly influence the magnitude of the effect size.

**Fig. 8:**
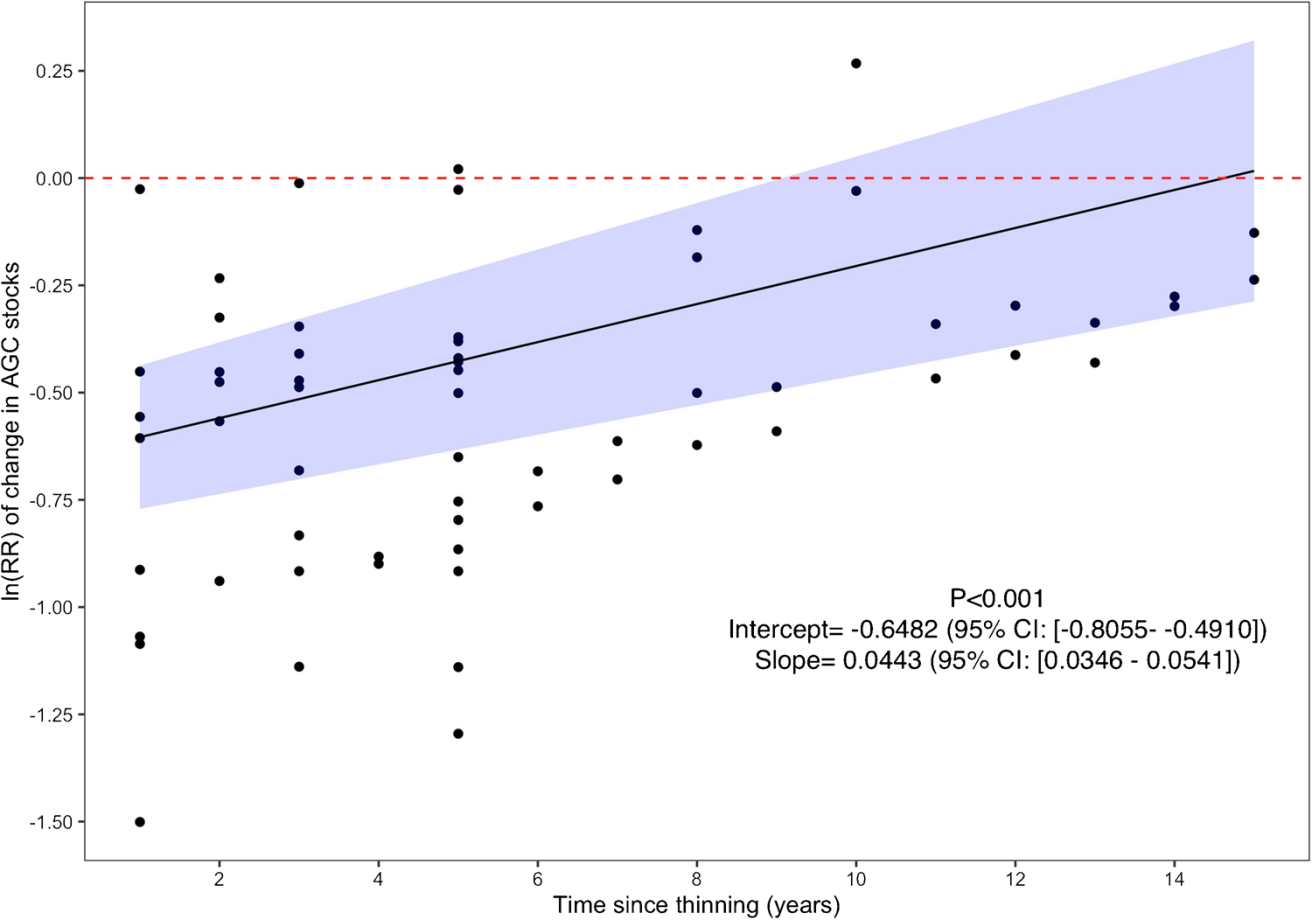
Change in the effect size of thinning operations as a function of time since thinning. The significance of the regression is indicated by the *p*-value in the lower right as well as the intercept and slope values with their corresponding 95% confidence interval. The area shaded in blue around the regression lines indicate the 95% confidence interval

When we inserted the “time since treatment” continuous moderator variable in the model, we found that as time since thinning increased, the negative effect of thinning on carbon lessened (*p*-value < 0.001) (Fig. 8). For every additional year since the treatment, the effect size of thinning on carbon stocks decreased by 4.6% (Fig. 8), suggesting that after 14 years the thinning effect disappears.

Mean annual precipitation also had a significant negative effect (*p*-value = 0.04) on the magnitude of the change in aboveground carbon (Figure S3.3). For every additional millimeter of precipitation received per year, the effect of thinning on carbon stocks was reduced by 0.03% (Figure S3.3). Finally, we found that as the percent of basal area removed increased, the negative effect size of thinning increased (*p* < 0.001) (Table S5.3). For every additional percent of basal area removed, the effect of thinning on carbon stocks was increased by 1.3% (Table S5.3).

## Discussion

Enhanced aboveground carbon sequestration in plantations via improved stand management is a prominent strategy for mitigating climate change. Our study quantified the effect size of three dominant silvicultural practices—intercropping N-fixing species, fertilization with inorganic NPK, and stand density management via thinning—on aboveground carbon stocks in plantations. We found that the magnitude of the effect size often depended on context and below we discuss the dominant drivers of variation and implications for landowners and land managers.

### Interplanting of N-Fixing Plants

We found that only some intercropping N-fixing species may be beneficial (in our case *Albizia*, *Enterolobium, Hippophae*, and *Leucaena* species) and/or primarily when the crop trees are mature. The species-specific effects may be mediated by compatibility of growth patterns [56, 57]. Growth compatibility can occur through at least three mechanisms: reduced competition for light via canopy stratification and crown complementarity, reduced competition for nutrients and water via root stratification, and direct or indirect growth facilitation [16, 58, 59]. All three mechanisms may explain our results. We found that intercropping with shade tolerant or moderately shade tolerant species (e.g., *Leucaena* spp., *Enterolobium* spp.) had greater effects on aboveground carbon relative to shade intolerant species (e.g., *Alnus* spp.). Root stratification couples trees with deep taproots (e.g., *Eucalyptus spp.*) that can acquire nutrients and water from lower soil horizons with species with shallow extensive horizontal root systems (e.g., *Acacia spp*.) [60]. Interplanting N-fixing plants may also confer benefits through chemical root stratification, which may facilitate mycorrhizal relationships between intercropped species and the main tree crops [16]. Furthermore, although we did not directly test these interactions, interplanting N-fixing legumes facilitate nutrient access for plantation trees by decreasing soil pH which promotes soil weathering and therefore general nutrient availability [61•]. Lastly, companion crops may inhibit the growth of competitors via allelopathic effects and/or eliminate local pathogens [58, 62].

Our results also showed that interplanting N-fixing plants become more beneficial through time. Early growth incompatibility and poor establishment of nitrogen-fixing companion crops could explain the negative or absent effect of intercropping early in the rotation [63, 64]. When crops are planted simultaneously, intense competition between them for resources such as light water or nutrients may emerge soon after their establishment [56, 65••]. Conversely, when the N-fixing species is established a few years after the main crop, the former tends to compete with the latter to a much lesser degree for light and water [56, 65••, 66, 67]. As a result, complementarity between the crops increases and the main crop can allocate a greater proportion of aboveground growth using the additional N provided by the companion crop [65••].

Our analysis also showed that several environmental, biological, and human factors influence the magnitude of response of the major crops to interplanting of N-fixing species. The main tree crop benefited more from the treatment when it was performed on wetter soils. Soil moisture significantly affects N-fixation by controlling nodulation and nitrogenase activity [68]. Moist soils promote nodulation and nitrogenase activity while drier soils reduce the number of nodules produced and inhibit nitrogenase activity, resulting in very low rates of nitrogen fixation [68]. Wetter soils also promote the growth of companion crops themselves, which allows for more N-fixation and thus increases the amount of available nitrogen in the soil [68].

Furthermore, interplanting N-fixing crops was most beneficial when it was performed in plantations growing on lands that previously hosted agricultural crops, compared to those previously under tree cover. Agricultural lands often experience soil impoverishment, especially when intense cropping techniques are used [69]. Low nutrient concentration, especially for N, appears to favor complementarity between the main tree crop and its N-fixing companion crop [65••]. Nonetheless, since soil impoverishment also depends on the intensity of the land use, which was not captured here, this result should be interpreted with caution.

### Inorganic NPK Fertilization

The use of NPK fertilizers significantly increased the aboveground carbon stocks of plantations, which aligns with the findings of others [70, 71]. However, once we accounted for the CO_2_e emissions resulting from inorganic N fertilizer manufacturing and use, we found that higher levels of fertilizer application could negate and/or overwhelm the increase in aboveground carbon stocks induced by fertilization. This highlights that the climate mitigation potential of fertilized stands could be substantially overestimated if fertilizer emissions are not taken into consideration. This also emphasizes that inorganic fertilizers have a greenhouse gas cost that can exceed their carbon benefit, particularly when they are applied in large amounts [72]. To maximize the climate benefits of using synthetic fertilizers in planted forests, a holistic accounting of greenhouse gas emissions associated with producing and applying fertilizers is therefore needed [73].

We found that the benefit of NPK fertilization decreased with time. This phenomenon has been noticed in previous studies and reviews [14, 74, 75] and several factors are believed to explain this trend. During the initial establishment phase, young seedlings generally do not have well developed root systems and providing easily accessible inorganic forms of nutrients can increase growth [18•, 74]. Over time, unfertilized seedlings develop their root systems and gain access to larger nutrient pools, which could explain the decrease in the magnitude of the effect size observed here [32, 74]. Furthermore, plantations trees increasingly rely on organic forms of N like glycine relative to inorganic forms (e.g., NH_3_ & NH_4_^+^) as they age [76], which could explain the lower effect size of fertilizers later in the rotation. Such a phenomenon might also apply in the context of the interplanting treatment discussed before. The effect of fertilizers on biomass carbon growth has also been suggested to decrease over time due to increased allocation of resources towards reproduction, which leads to lower nutrient uptake rates [77].

Our results showed that the benefit of NPK fertilization on growth declined as precipitation increased. High precipitation levels increase soil water content and, in some cases, push it beyond field capacity (i.e., soil becomes saturated) [78]. Saturated soils tend to be highly prone to leaching of very mobile nutrients such as N, which reduces their availability for plant uptake [15••, 78]. Our results also suggest that plantations growing on soils with udic moisture regimes experienced the largest increases in aboveground carbon when fertilized. Nutrients are generally more available when soil moisture levels are optimum and remain adequate for most of the year, as is the case for soils with an udic soil moisture regime [79].

Conversely to interplanting, the benefit of NPK fertilizers was higher when they were applied in plantations growing on lands that previously hosted tree plantations or natural forests. Harvested lands often experience soil impoverishment, especially when the whole tree harvesting method is employed [80, 81]. Indeed, harvest promotes nutrient mineralization and nitrification, making them more mobile and therefore more subject to leaching [82]. The removal of trees themselves also leads to a decrease in soil nutrient capital and can curb the growth of the next trees [83]. Fertilization is often used to compensate for the loss of nutrients [83], and poor soil post-harvest may explain the fertilizer benefit we observed. However, since this analysis did not account for the effect of land use intensity on soil impoverishment, this result should be interpretated with caution. Overall, these findings related to site conditions underline the fact that response of trees to fertilization is heavily site dependent and varies with site productivity and quality, and/or intensity of prior land use.

This study also revealed that plantation stands store more aboveground carbon when fertilizer applications are performed in pulses, rather than continuously. Aber et al. [84] and Saarsalmi and Mälkönen [85] found similar results and attributed it to higher nutrient-use efficiency under pulse fertilization. Furthermore, several studies have stressed that continuous application of N favors faster N saturation, which reduces the positive effects of inorganic N-based fertilizers on biomass growth in the long-term [86]. Since N saturation is also believed to promote nitrate leaching from soils [87], increase emissions of N_2_O [84], and induce large reduction in mycorrhizal symbionts [88], our results underline that careful handling of fertilizer operations is needed to maximize tree growth while minimizing negative environmental side effects. Nonetheless, this difference in growth responses to pulsed and continuous fertilization might also be caused by other chemical or physical soil variables (e.g., initial soil fertility, soil pH) which were not captured in this analysis.

### Thinning

Our results showed that thinning operations significantly lowered aboveground carbon in plantations, which aligns with the findings of others [40, 89]. Given that thinning is performed to reallocate resources and growing space to target trees of primary value and induces a trade-off in loss of stand-level carbon, this result was expected. Indeed, increased growth rates for individual trees post-thinning have been well documented [44, 90, 91]. We also identified an attenuation of the effect on overall stand aboveground carbon with increasing time since thinning (Fig. 8). Our results suggest that at the stand level, the negative effect of thinning on aboveground carbon lasts for approximately a decade before becoming attenuated.

Furthermore, we found that the negative effect of thinning on aboveground carbon might be amplified when performed in stands located in dry areas and growing on xeric soils. Non-thinned trees growing in stands on mesic and wetter soils tend have access to more resources such as water and nutrients after treatment than the same trees growing on xeric soils, which could therefore induce a greater growth response to thinning operations and a weaker treatment effect in the former scenario than in the latter.

Thinning operations appear to have a more negative effect on stand carbon when they are performed on former agricultural lands. Nutrient and water availability are generally lower in those areas, and fertilization and irrigation are often needed to ensure afforestation/reforestation success [92]. As a result, thinned stands growing on former agricultural lands might have access to smaller nutrient and water pools that constrain post-thinning growth and therefore induce a larger thinning effect on stand-level aboveground carbon. Furthermore, former agricultural lands tend to be more compacted due to heavy machinery use [93], which could restrict the common increase in resource availability after thinning operations and further accentuate the effect on aboveground carbon [43]. Overall, these results stress that response of stands to thinning is heavily site dependent. Stands on more productive sites will often respond quite differently to the same thinning treatment than a stand on lower quality sites [94].

### Caveats and Potential Limitations

Our study focuses on aboveground live tree carbon stocks only and did not examine changes in belowground carbon and soil organic carbon in response to fertilization and thinning. Belowground carbon and soils are substantial carbon pools in forest ecosystems. For example, soil carbon stocks are believed to represent 90% and 50% of the total carbon stock in boreal and tropical forests respectively [95]. Furthermore, in these forest types, roots are believed to store between 23 and 27% of total tree biomass carbon [96]. However, we omitted these pools due to little data availability and inconsistent field sampling methods across reviewed studies. This decision was further justified by the fact that changes in soil carbon stocks tend to be small relative to changes in the aboveground carbon pool and it takes longer to detect them [97]. Previous studies have found positive effects of NPK fertilization and interplanting N-fixing plants on soil carbon stocks in forest plantations [86, 98, 99]. Other studies have found neutral or negative effects of thinning on soil carbon stocks [35, 36, 40, 89, 100] and negative effects on belowground carbon as well [100]. If these results occur in other forest plantation management studies, focusing solely on carbon in aboveground biomass tissues may underestimate the impact of forest management actions such as fertilization on total ecosystem carbon stocks and overestimate it in the context of thinning. Additional empirical data on the impact of these management operations on plantations total carbon stocks as well as studies reporting responses of the different carbon pools are needed.

Data limitations also partly restricted our ability to assess the exact timing at which the positive effects of inorganic fertilizers on aboveground stand carbon stocks disappear. Although the data stopped at 5 years since treatment, if the decline through time continues following the same path, then we would predict the carbon benefits of inorganic fertilizers to disappear eight years after treatment. Further data on the impact of time on the effect size of fertilizers would therefore be valuable to refine our predictions. Moreover, data were heavily skewed towards younger ages across all of our focal silvicultural treatments. Long-term measurements of the effects of silvicultural treatments on plantation forest carbon stocks will greatly improve our understanding of the climate mitigation potential of these systems.

### Management Implications

This study reveals that the use of N-fixing companion crops as a fertilization technique is challenging and primarily depends heavily on growth complementarity between the crop and intercropped species. Knowledge of the silvics of individual species is a critical precursor to ensure successful growth benefits, and poorly implemented systems may induce null effects on growth. However, use of intercropping as the main fertilization technique can also reduce N_2_O emissions and nutrient volatilization, which are common issues associated with the application of inorganic NPK fertilizers [101]. Factors such as prior land use, soil moisture regime, and method of implementation are also likely to be key moderators of the effect of interplanted N-fixing plants on aboveground carbon. Knowledge of the site characteristics and history, as well as interactions between tree species seem therefore crucial to ensure successful associations.

Inorganic fertilizers, despite being growth catalyzers, can have detrimental effects on the environment which includes water eutrophication via nutrient leaching and runoffs, and air pollution via greenhouse gas (GHG) emissions from fertilizers manufacturing and nutrient volatilization. Thus, there are benefits to minimizing additions. Our results show that NPK fertilizers are not universally beneficial and their positive effects, when observed, decline through time. Furthermore, we found that GHG emissions resulting from N fertilizer manufacturing and in-field application could exceed the aboveground carbon gains induced by their use. Our results also stress that factors such as prior land use and soil moisture regime may be key moderators of the effect of fertilizers on aboveground carbon. Our findings emphasize therefore the need to apply the right rate of nutrients at the right times and in the right context. Landowners should carefully handle fertilization operations to maximize their carbon benefits and minimize their costs.

Next, our study suggests that the initial decrease in aboveground carbon caused by thinning operations could be compensated over time by the recovery of carbon post-thinning, particularly when coupled with extended rotation lengths. Indeed, by increasing rotation lengths, landowners can further attenuate the effects of thinning on stand carbon up to a point at which the initial loss of carbon is offset by the recovery of a productive and well-stocked stand [40, 42, 102]. From there, the individual trees in thinned stands could sequester additional carbon to levels as high or higher than unthinned stands of the same age [42, 102]. Critically, the likelihood of recovering unthinned carbon levels post-thinning will depend on the intensity of thinning and thinning technique employed, with more intensive thinning practices tending to result in longer or unachievable recovery of aboveground carbon [15••, 42, 91, 102]. For example, plots thinned from below may rapidly recover carbon sequestration rates equivalent to control plots, while stands that experienced dominant and crown thinning operations may have longer periods to carbon recovery [42]. However, thinning can also confer important forest structural qualities important for management of forest carbon beyond maximization of standing aboveground carbon stocks [13••], such as conferring resistance to low or medium-intensity wildfire in fire-prone landscapes and insect/pathogen outbreaks [13••, 103]. Further research on these co-benefits and the coupling of thinning with extended rotations is needed to validate the use of thinning as a forest carbon management tool.

Lastly, this meta-analysis underlines the need for forestry practitioners to be aware of site conditions, quality, and land use history before conducting thinning operations because their effects can vary from site to site, and they tend to strongly depend on local conditions. Additional studies on the influence of soil moisture regimes and previous land use on the magnitude of the effect size of thinning on stand aboveground carbon are needed to consolidate our results. Overall, our study underscores that selecting the appropriate species and treatments for each site is crucial to ensure an effective carbon management plan in forest plantations.

## Conclusion

Improved forest management has been highlighted as a key natural climate solution because of its ability to deliver climate benefits within short-time scales [1, 5, 7•, 8•]. Nonetheless, there is still substantial uncertainty regarding the forest practices that would help realize this mitigation potential and the context in which they would deliver the most climate benefits [1]. Our study provides additional considerations that help facilitate the use of improved management practices to increase plantations carbon stocks and hence mitigate climate change. By specifying the conditions in which fertilization and stand density management tend to be the most beneficial for carbon storage purposes, this study provides additional information to forest practitioners on how to use them as carbon management tools. Although not all management actions studied here provide substantial increases in carbon storage over the entire lifetime of plantation trees (e.g., interplanting N-fixing trees), they still may be desirable by enhancing monocultures biodiversity levels and resilience to disturbances such as pests or natural disasters [18•, 104, 105]. The latter tends to be crucial for the permanence and durability of carbon sequestered on the landscape, particularly under a changing climate [106•].

## Supplementary Information

Below is the link to the electronic supplementary material.

Supplementary file1 (DOCX 3.79 MB)

## Data Availability

Data used for the study are published on Zenodo (10.5281/zenodo.7789868)
