# Supplementary material for: Quantifying the Effect Size of Management Actions on Aboveground Carbon Stocks in Forest Plantations
Source: Curr For Rep. 2023 Apr 11;9(3):131–48. doi: 10.1007/s40725-023-00182-5 (PMC10328870; doi:10.1007/s40725-023-00182-5)
Supplement: Supplementary file 1 — Supplementary file1 (DOCX 3.79 MB) [file 40725_2023_182_MOESM1_ESM.docx]

**SUPPLEMENTARY INFORMATION**

**“Quantifying the effect size of management actions on aboveground carbon stocks in forest plantations”**

Cyril H. Melikov, Jacob J. Bukoski, Susan C. Cook-Patton, Hongyi (Stella) Ban, Jessica L. Chen, Matthew D. Potts

*S1 | Studies included in this study*

**Table S1.1**: Studies and species used in the meta-analysis on the effect of intercropping N-fixing plants on plantations biomass. The numbers in parenthesis (e.g (1:1)) indicate the tree crop:intercropping ratio in the stand, if the study provided them.

| Author | Species | Genus | Country | Latitude | Longitude | Intercropped N-fixing plant | Age |
| --- | --- | --- | --- | --- | --- | --- | --- |
| Austin et al. 1997 | *Eucalyptus grandis* | *Eucalyptus* | USA (Hawaii) | 21.3453 | -157.7214 | *Leucaena leucocephala, Paraserianthes falcataria, Enterolobium cyclocarpum* | 1,2,4 |
| Baliero et al. 2002 | *Eucalyptus grandis* | *Eucalyptus* | Brazil | 37.7833 | 127.8000 | *Albizia guachepele* | 5 |
| Beets and Whitehead, 1996 | *Pinus radiata D. Don* | *Pinus* | New Zealand | -23.0333 | -48.6333 | *Lupinus arboreus* | 9,14 |
| Binkley et al. 2003 | *Eucalyptus saligna* | *Eucalyptus* | USA (Hawaii) | 19.5000 | -155.2500 | *Falcataria moluccana* | 3,7,10,18,20 |
| Bogdan et al. 2009 | *Alnus glutinosa* | *Alnus* | Croatia | 45.6615  46.0100 | 17.50720  17.17050 | *Salix spp.* | 14,16 |
| Bouillet et al. 2016 | *Eucalyptus grandis x urophylla* | *Eucalyptus* | Brazil | -19.2667  -23.1833  -23.1833 | -41.7833  -48.4167  -48.4167 | *Acacia mangium* | 1.83,2.25,2.5,2.58,2.83, 6,6.08,6.23,6.25,6.42 |
| Chen, 2009 | *Cunninghamia lanceolata* | *Cunninghamia* | China | 26.6540 | 118.1610 | *Alnus cremastogyne* | 10 |
| DeBell et al. 1997 | *Eucalyptus saligna* | *Eucalyptus* | USA (Hawaii) | 19.8978 | -155.1270 | *Albizia falcataria* | 2,4,6,8,10 |
| Epron et al. 2013 | *Eucalyptus grandis Hybrid (Eucalyptus grandis x Eucalyptus urophylla)* | *Eucalyptus* | Brazil  Congo | -23.0333  4.7333333 | -48.6333  12.0167 | *Acacia mangium Acacia mangium* | 6,7 |
| Forrester et al. 2004 | *Eucalyptus globulus* | *Eucalyptus* | Australia | -37.8744 | 146.9115 | *Acacia mearnsii* | 11 |
| Forrester et al. 2007 | *Eucalyptus globulus* | *Eucalyptus* | Australia | -37.5833 | 149.1667 | *Acacia mearnsii (1:1)* | 10 |
| Forrester et al. 2010 | *Eucalyptus globulus* | *Eucalyptus* | Australia | -37.5833 | 149.1667 | *Acacia mearnsii (1:1)* | 15 |
| Fredericksen et al. 1993 | *Pinus taeda* | *Pinus* | USA | 37.2239 | -80.4224 | *Robinia pseudoacacia* | 3 |
| Ghorbani et al. 2018 | *Populus deltoides* | *Populus* | Iran | 36.4774 | 52.1167 | *Alnus subcordata (1:1) Alnus subcordata (3:1)* | 3,4,8,20 |
| Groninger et al. 1997 | *Pinus taeda* | *Pinus* | USA | 37.2239 | -80.4224 | *Robinia pseudoacacia* | 3,4,5 |
| Han et al. 2008 | *Eucalyptus grandis* | *Eucalyptus* | China | 24.7750 | 107.7333 | *Acacia mangium* | 4 |
| Kaye et al. 2000 | *Eucalyptus saligna* | *Eucalyptus* | USA | 19.5000 | -155.2500 | *Albizia falcataria* | 17 |
| Laclau et al. 2008 | *Eucalyptus grandis* | *Eucalyptus* | Brazil | -22.7667 | -43.6833 | *Acacia mangium (2:1)* | 0.5, 1, 1.5,2.5 |
| le Maire et al. 2013 | *Eucalyptus grandis* | *Eucalyptus* | Brazil | 4.7333 | 12.0167 | *Acacia mangium* | 1,2,3,4,5,6 |
| Li et al. 2013 | *Eucalyptus camaldulensis* | *Eucalyptus* | China | 25.6686 | 101.8586 | *Leucaena leucocephala* | 20 |
| Liu et al. 2015 | *Casuarina equisetifolia* | *Casuarina* | China | 20.0478 | 110.7269 | *Acacia mangium (1:1) Acacia mangium (3:1)* | 10 |
| Liu, 2017 | *Cunninghamia lanceolata* | *Cunninghamia* | China | 24.9330 | 118.0000 | *Betula luminifera* | 5,10,15 |
| Mao et al. 2010 | *Populus x xiaozhuanica* | *Populus* | China | 41.3083 | 119.6000 | *Hippophae rhamnoides* | 5,15 |
| Marron et al. 2018 | *Populus x euramericana* | *Populus* | France | 47.8071 | 1.9767 | *Robinia pseudoacacia* | 1.42,2.33,3.33,4.25 |
| Mayoral et al. 2017 | *Anacardium excelsum* | *Anacardium* | Panama | 9.2167 | -79.7833 | *Dalbergia retusa* | 2,7 |
| Moore et al. 2011 | *Pseudotsuga menziesii* | *Pseudotsuga* | United States | 44.2261 | -122.1931 | *Alnus rubra* | 15 |
| Oliviera et al. 2018 | *Populus alba* | *Populus* | Spain | 40.4667 | -3.3667 | *Robinia pseudoacacia (25%)* | 3 |
| Pan et al. 1998 | *Eucalyptus camaldulensis x tereticornis* | *Eucalyptus* | China | 23.5000 | 112.1667 | *Acacia auriculiformis* | 5 |
| Parotta, 1999 | *Casuarina equisetifolia Eucalyptus robusta* | *Casuarina, Eucalyptus* | USA (Puerto Rico) | 18.4373 | -66.2579 | *Leucaena leucocephala Leucaena leucocephala* | 4 |
| Redondo-Brenes et al. 2006 | *Vochysia guatemalensis* | *Vochysia,* | Costa Rica | 10.4306 | -84.0069 | *Callophylum brasiliense* | 13 |
| Santos et al. 2016 | *Eucalyptus grandis x urophylla* | *Eucalyptus* | Brazil | -22.7600 | -43.6700 | *Acacia mangium* | 2.5,5 |
| Schweier et al. 2019 | *Populus maximowiczii x trichocarpa* | *Populus* | France | 48.0167 | 7.0167 | *Robinia pseudoacacia* | 2 |
| Son et al. 2007 | *Pinus koraiensis* | *Pinus* | South Korea | -23.0333 | -48.6333 | *Alnus hirsuta* | 28 |
| Toïgo et al. 2021 | *Pinus pinaster* | *Pinus* | France | 44.7333 | -0.7667 | *Betula pendula* | 7 |
| Weng, 2008 | *Cunninghamia lanceolata* | *Cunninghamia* | China | 27.4721 | 117.1914 | *Ormosia hosiei* | 14 |
| Xiao et al. 1999 | *Eucalyptus leizhouensis* | *Eucalyptus* | China | 20.8772  20.6987 | 109.8316  110.0343 | *Acacia crassicarpa* | 6 |
| Xu, 1998 | *Pinus elliotti* | *Pinus* | China | 23.6090 | 117.4090 | *Casuarina equisetifolia* | 6 |
| Yang et al. 2009 | *Eucalyptus urophylla* | *Eucalyptus* | China | 20.3500 | 110.0170 | *Acacia crassicarpa* | 6 |
| Yang, 2015 | *Eucalyptus spp.* | *Eucalyptus* | China | 25.0260 | 116.9960 | *Acacia concinna* | 6 |
| Ye, 2002 | *Pinus elliottii* | *Pinus* | China | 23.5830 | 117.0330 | *Casuarina equisetifolia* | 7 |
| Ye, 2013 | *Pinus elliottii* | *Pinus* | China | 25.3230 | 118.3200 | *Acacia mangium* | 5 |
| Zhang and Chen, 2007 | *Pinus tabuliformis* | *Pinus* | China | 37.5917 | 111.3750 | *Hippophae rhamnoides* | 21 |
| Zou, 2006 | *Pinus massoniana* | *Pinus* | China | 25.4833 | 118.3833 | *Acacia dealbata* | 7 |

**Table S1.2**: Studies and species used in the meta-analysis on the effect of inorganic NPK fertilization on plantations biomass

| Author | *Species* | *Genus* | Location | Latitude | Longitude | Treatments (Total amount of fertilizer added) | Fertilizer application methodology | Age |
| --- | --- | --- | --- | --- | --- | --- | --- | --- |
| Beets and Whitehead, . 1996 | *Pinus radiata* | *Pinus* | New Zealand | -38.4333 | 176.2167 | Control  NPK (962 kg N/ha, 407 kg N/ha, 412 kg N/ha) | Continuous (for the first 10 years) | 9,14 |
| Bennett et al. 1997 | *Eucalyptus globulus* | *Eucalyptus* | Australia | -38.2667 | 146.0500 | Control  NPK (400kg N/ha, 200kg P/ha, 200 kg K/ha) | Pulse | 3 |
| Gholz et al. 1991 | *Pinus elliottii* | *Pinus* | USA | 29.7333 | -82.1583 | Control  NPK (360 kg N/ha, 140 kg P/ha, 280 kg K/ha) | Continuous | 21,22,23 |
| Ingerslev and Hallbäcken 1999 | *Picea abies* | *Picea* | Denmark | 56.4889 | 8.3578 | Control  NPK (120kg N /ha, 230kg P/ha, 140kg K/ha) | Pulse | 59 |
| Jaquetti and Gonçalves, 2017 | *Inga edulis*  *Schzilobium amazonicum*  *Dypteryx odorata* | *Inga*  *Schzilobium*  *Dypteryx* | Brazil | -1.9442 | -59.4181 | Control  NPK (45kg N/ha, 157.5 kg P/ha, 82.5 kg K/ha)  NPK (1067.5 kg N/ha, 92.5 kg P/ha, 411.25 kg K/ha)  NPK (1112.5 kg N/ha, 250 kg P/ha, 492.5 kg K/ha) | Pulse | 1 |
| Jokela and Martin, 2000 | *Pinus eliottii Pinus taeda* | *Pinus* | USA | 29.5000 | -82.3333 | Control  NPK (360 kg N/ha, 143 kg P/ha, 317 kg K/ha) | Continuous | 13 |
| Madeira et al. 2002 | *Eucalyptus globulus* | *Eucalyptus* | Portugal | 39.3333 | -9.2167 | Control  NPK (734kg N/ha,251kg P/ha; 660 kg K/ha  Control (46kg N/ha, 26kg P/ha; 137kg K/ha)  NPK (952kg N/ha, 347kg P/ha, 917kg K/ha) | Continuous | 6 |
| Maier et al. 2004 | *Pinus taeda* | *Pinus* | USA | 34.9056 | -79.4833 | Control  NPK (586kg N/ha, 145kg P/ha337kg K/ha) | Continuous | 12 |
| Resh et al. 2003 | *Eucalyptus nitens* | *Eucalyptus* | Australia | -42.8167  -42.4833 | 147.6000  146.4667 | Control  NPK (900 KgN/ha; 400 Kg P/ha) | Pulse | 8 |
| Ryan et al. 2004 | *Eucalyptus saligna* | *Eucalyptus* | USA (Hawaii) | 19.8411 | -155.1244 | Control  NPK (1610 kg N/ha, 750 kg P/ha, 1180kg K/ha)  NPK (830 kg N/ha; 378 kg P/ha, 628 kg K/ha) | Continuous | 0.5, 1,2,3,4,5,6 |
| Samuelson et al.2004 | *Pinus taeda* | *Pinus* | USA | 30.8000 | -84.6500 | Control  NPK (379.158 kg N /ha, 218kgP/ha; 510kg K/ha) | Continuous | 2,3,4,  5,6 |
| Shan et al 2001. | *Pinus eliottii* | *Pinus* | USA | 30.5667 | -81.8333 | Control  NPK (224 kg di-ammonium phosphate/ha at 1 years old) + NPK (280kg di-ammonium phosphate/ha; 280 kg Urea /ha, 228 kg KCL/ha at 12 years old) | Pulse | 17 |
| Shujauddin and Kumar, 2003 | *Ailanthus triphysa* | *Ailanthus* | India | 10.2167 | 76.2167 | Control  NPK (150 kg N/ha, 75kg P/ha/yr, 75 kg K/ha/y) NPK (300 kg N/ha, 150 kg P/ha, 150 kg K/ha) NPK (450 kg N/ha, 225kg P/ha, 225 kg K/ha) | Pulse | 9 |
| Sicard et al. 2006 | *Picea abies* | *Picea* | France | 47.3028 | 4.0678 | Control  NPK (5.4 kg N/ha; 96 kg P/ha, 20 kg K/ha) | Pulse | 28 |
| Stape et al. 2010 | *Clonal Eucalyptus* | *Eucalyptus* | Brazil | -22.3500  -21.5333  -19.8167  -18.5833  -18.0333  -17.3333  -16.3500 | -46.9667  -48.3667  -40.0833  -42.9833  -39.8667  -43.8333  -39.5667 | Control  NPK (724kg N/ha, 284kg P/ha,669kg K/ha) NPK (594kg N/ha, 240kg P/ha,546kg K/ha) NPK (21kg N/ha, 33kg P/ha,136kg K/ha) NPK (86kg N/ha, 40kg P/ha,171kg K/ha)  NPK (732kg N/ha, 145kg P/ha,570kg K/ha) | Continuous | 1,2,3,  4,5,6 |
| Subedi et al. 2014 | *Pinus taeda* | *Pinus* | USA | 29.5000 | -82.3333 | Control  NPK (120kg/ha N, 53kg/ha P, 99kg/ha K) | Continuous | 1,2,3 |
| Susanto 2017 | *Maranga gigantea* | *Maranga* | Indonesia | -0.5018 | 117.1393 | Control  NPK (4.32 kg N/ha, 4.32 kg P/ha, 4.32kg K/ha)  NPK (8.48 Kg N/ha, 8.48 kg P/ha, 8.48 kg K/ha)  NPK (12.8 kg N/ha, 12.8 kg P/ha, 12.8 kg K/ha)  NPK (17.1 kg N/ha, 17.1 kg P/ha, 17.1 kg K/ha) | Pulse | 1 |
| Vogel et al. 2015 | *Pinus taeda* | *Pinus* | USA | 29.7494 | -82.2175 | Control  NPK (760kg N/ha, 180kg P/ha, 120kg K/ha)  NPK (450kg N/ha, 100kg P/ha, 150kg K/ha) | Pulse | 10,11 |

**Table S1.3**: Studies and species used in the meta-analysis on the effect of thinning on plantations biomass

| Author | Species | Genus | Location | Latitude | Longitude | Treatment (% of original basal area removed) | Age |
| --- | --- | --- | --- | --- | --- | --- | --- |
| Chang and Preston, 2000 | *Thuja plicata Tsuga heterophyllaa Picea sitchensis* | *Thuja Tsuga Picea* | Canada | 50.6000 | -127.2500 | Control  Low thinning | 9 |
| Cheng et al. 2017 | *Cunninghamialanceolata* | *Cunninghamia* | China | 29.1500 | 118.4166 | Control  Moderate thinning (36% BA removed)  Heavy thinning (51% BA removed) | 22 |
| Forrester et al. 2012 | *Eucalyptus nitens* | *Eucalyptus* | Australia | -38.3833 | 146.6833 | Control  Thinned from 900 trees/ha to 300 trees/ha (67% BA removed) | 8.1 |
| Gresham et al. 2002 | *Pinus taeda* | *Pinus* | USA | 33.6094  33.6178 | -79.9775  -79.2231 | Control  Low thinning | 10 |
| Hennessey et al. 2004 | *Pinus taeda* | *Pinus* | USA | 33.9667 | -94.5833 | Control  Thinned to 25% of the original basal area (75% BA removed) Thinned to 50% of the original basal area (50% BA removed) | 10,11,12,1314,15,16,17, 18,20,21,22,23,24 |
| Kim et al. 2009 | *Pinus densiflora* | *Pinus* | South Korea | 35.4844 | 127.9711 | Control  Removed 541 trees/ha (41% BA removed) | 40, 41 |
| Kunhamu et al. 2009 | *Acacia mangium* | *Acacia* | India | 11.35833 | 76.3639 | Control  Low intensity thinning (33% BA removed)  Medium intensity thinning (50% BA removed) High intensity thinning (67% BA removed) | 9 |
| Ruiz-Mirazo et al. 2013 | *Pinus halepensis* | *Pinus* | Spain | 37.3833 | -3.0500 | Control  Light-random thinning (54 % BA removed) Light regular thinning (54% BA removed) Medium-random thinning (77 % BA removed) Medium-regular thinning (77% BA removed) Heavy-random thinning (86% BA removed) Heavy-regular thinning (86% BA removed) | 11, 13,15 |

*S2 | Description of soil moisture regimes.*

The soil moisture regime is defined by “the number of consecutive or cumulative days that the soil is either moist or dry in the moisture control section” and is determined based on the seasonal distribution of rainfall in what are so-called normal years [1]. Buol [2] defined normal years as “plus or minus 1 standard deviation of long-term mean annual precipitation.” In this context, the long-term refers to 30 years. Soils having a perudic soil moisture regime are water-saturated and found where precipitation surpasses evapotranspiration in every month [1,3]. As a result, perudic soils are always humid. The udic moisture regime is common to soils in humid regions having well-distributed rainfall events throughout the year [1,4]. Soils with udic moisture regimes receive enough rain during the summer that the amount of water already stored added to the amount of water received by rainfall exceeds the evapotranspiration rate [2,4]. The moisture control section of udic soils tends to be humid for as much as 90 consecutive days [1]. The perudic regime is often considered as a type of udic regime [3]. Soils with an ustic moisture regime are moisture-limited but soil moisture tends to be available when crop growing conditions are optimal (USDA, 2015). Ustic soils are found in sub-humid and sub-arid climates [3]. A soil moisture regime is classified as ustic when the amount of water stored in soils plus what is received by precipitation is smaller than the amount of water lost by soils via evapotranspiration [1,4]. The xeric soil moisture regime is most commonly found in areas with a Mediterranean climate with dry and warm summers and wet and cool winters [3]. This regime is classified as a dry regime and soils having this moisture regime are dry for 45 consecutive days following the summer solstice and wet for 45 consecutive days following the winter solstice [1].

*S3 | Supplementary Figures*

**Figure S3.1:** Change in the fertilizing effect size of NPK fertilizers as a function of precipitation levels. The significance of the regression is indicated by the p-value in the upper right as well as the intercept and slope values with their corresponding 95% confidence interval.


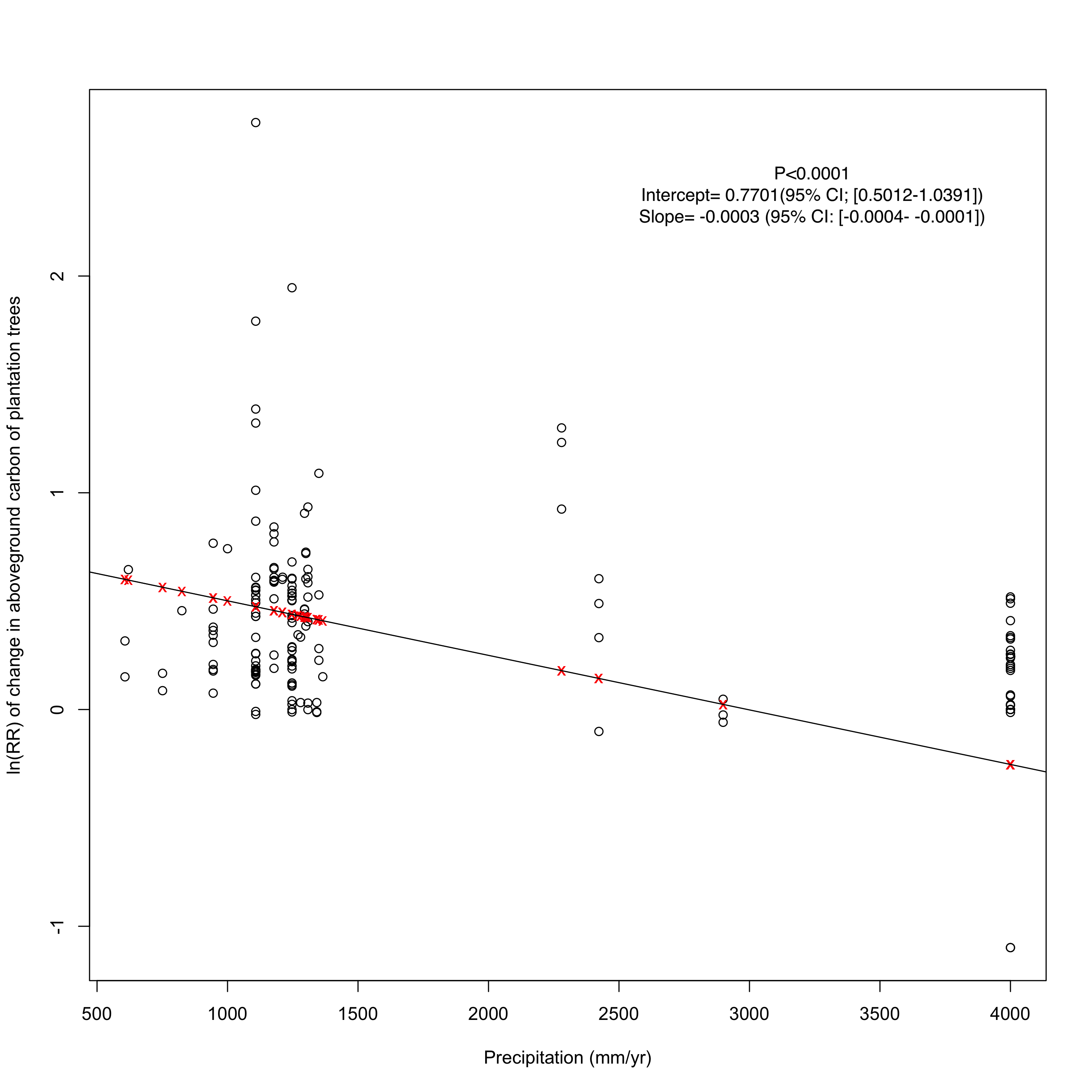


**Figure S3.2.** Net carbon balance of fertilized stands across studies. If the net carbon balance is positive, the fertilized stand is a net carbon sink while if the former is negative, the fertilized stand is a net carbon source. The red vertical dashed line corresponds to a net carbon balance of zero. Blue points are the individual data measurements.

*
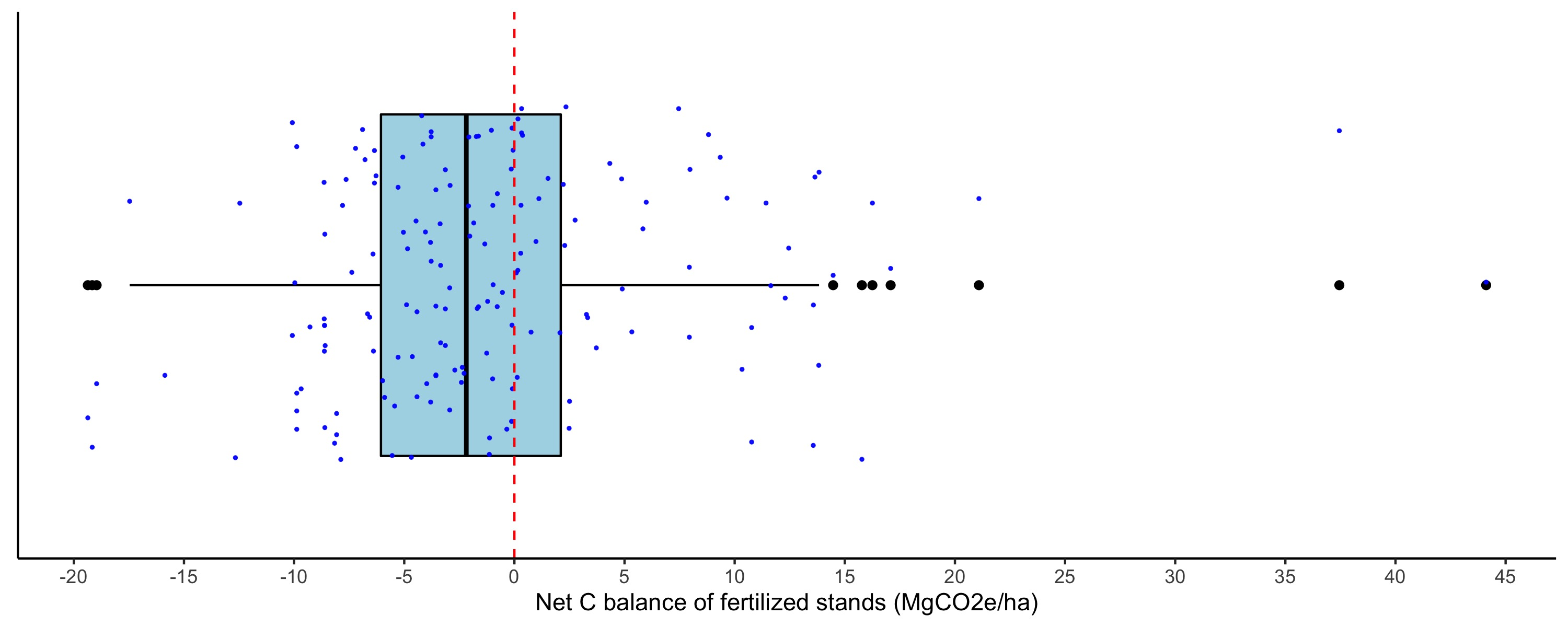
*

**Figure S3.3:** Change in the effect of thinning treatment on stands aboveground carbon levels as a function of mean annual precipitation levels. The significance of the regression is indicated by the p-value in the lower right as well as the intercept and slope values with their corresponding 95% confidence interval.

*
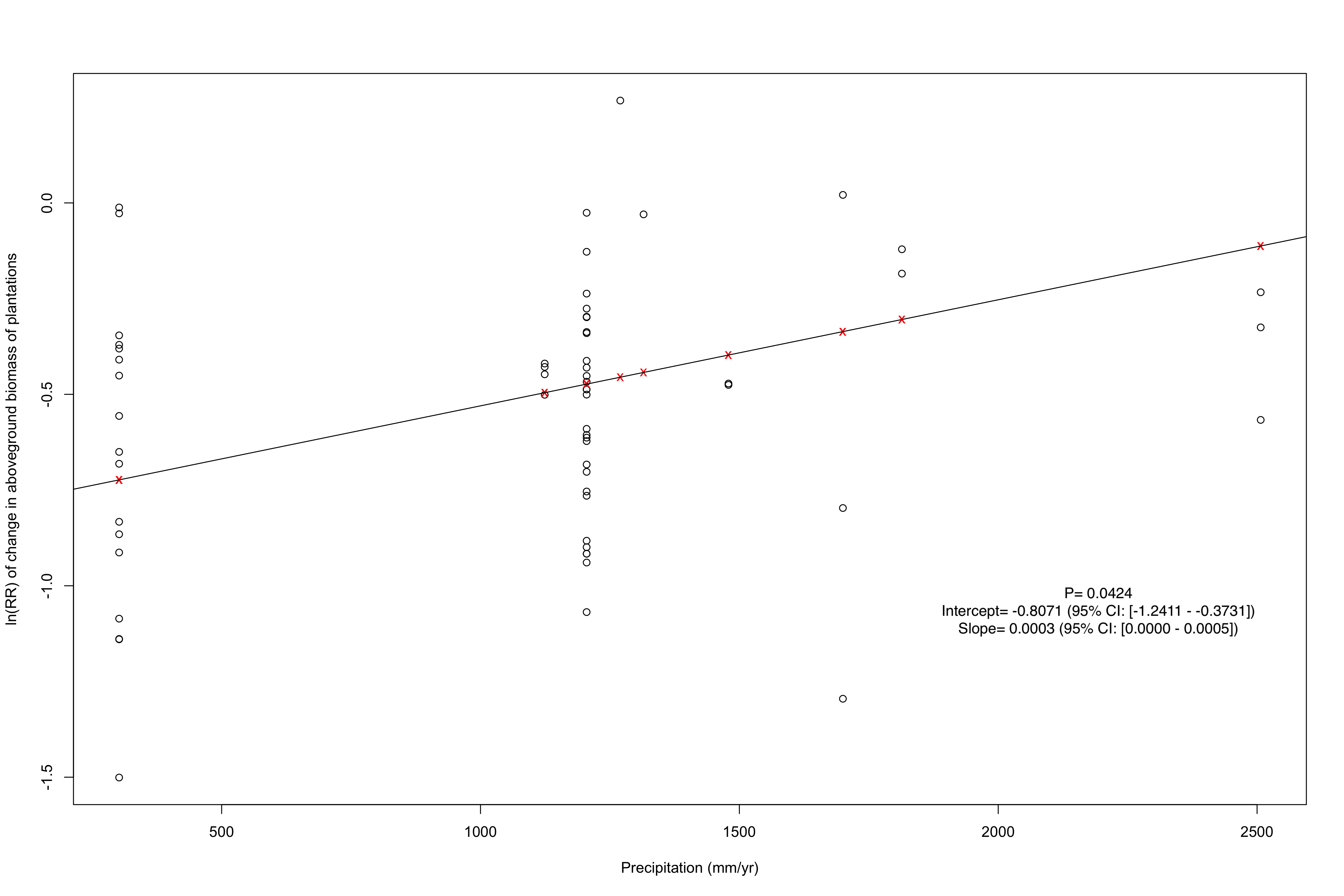
*

*S4 | Sensitivity Analysis*

To assess the sensitivity of our results to the imputed standard deviations, we performed a sensitivity analysis. We calculated the coefficient of variations (ratios of standard deviations and means) of treatment and control groups (interplanted vs pure or fertilized vs not fertilized) for all trials that reported them. For each set of ratios (coefficient of variations), we calculated the median of its distribution, then the first quartile (Q1) and the third quartile (Q3). Then, we replaced the missing standard deviations in the original dataset with imputed standard deviations using the median, then the first quartile, and finally the third quartile of the coefficient of variation distribution for the treatment or control groups. As a result of this step, we produced three different datasets. Each dataset we analyzed separately following the methods described in the manuscript. We then plotted the results produced by each dataset, together for each treatment (interplanting of N-fixing plants, inorganic NPK fertilization, thinning) (Fig. S4.1 & Fig. S4.2 & Fig. S4.3). We concluded that the results of the analysis were robust when the mean and the 95% confidence intervals changed marginally with changing imputed standard deviations. Conversely, we interpreted results with caution when the mean and 95% confidence intervals varied significantly with imputed standard deviations


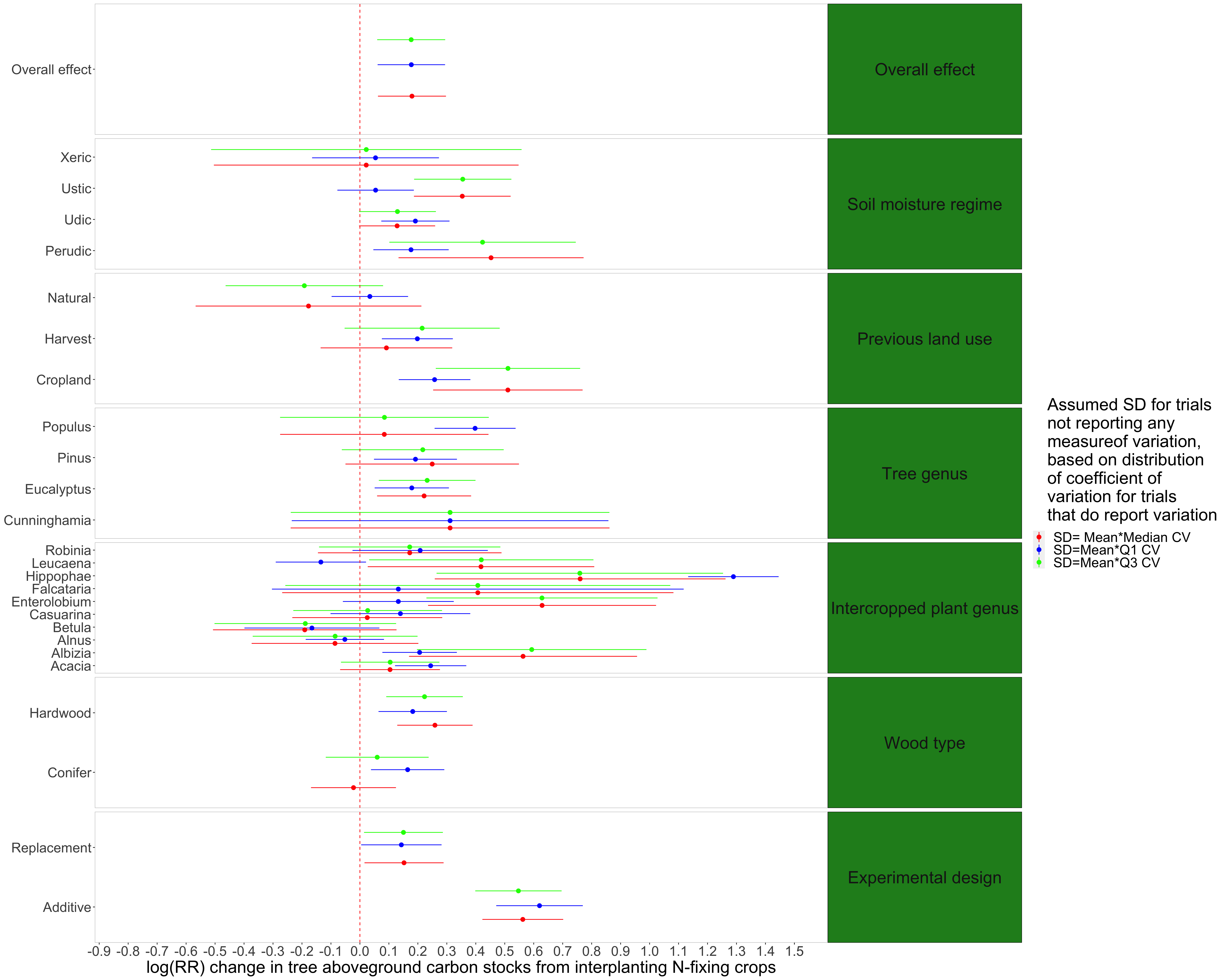


**Figure S4.1:** Sensitivity analysis of intercropping N-fixing plants meta-analysis to imputing missing standard deviations


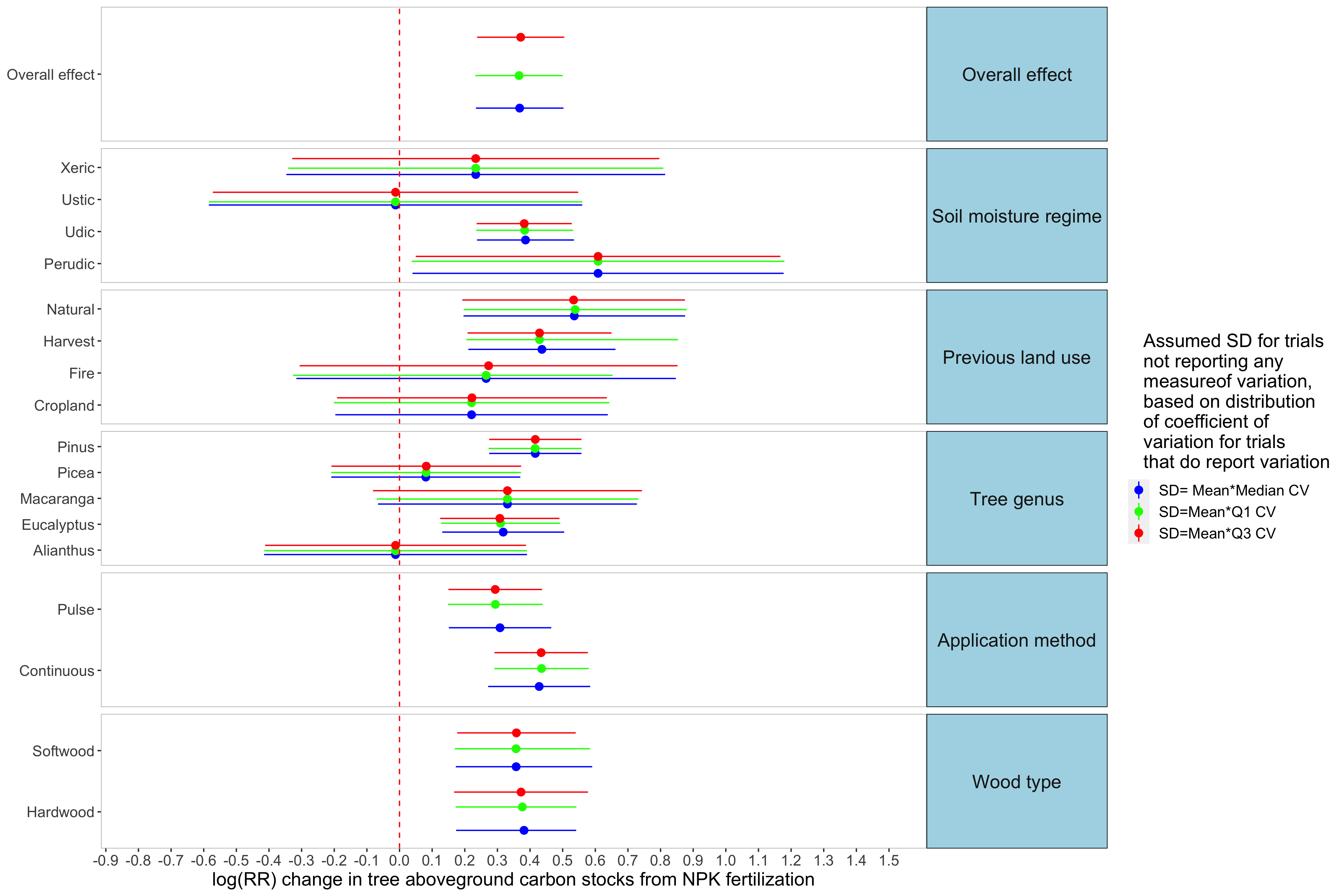


**Figure S4.2:** Sensitivity analysis of NPK fertilization meta-analysis to imputing missing standard deviations


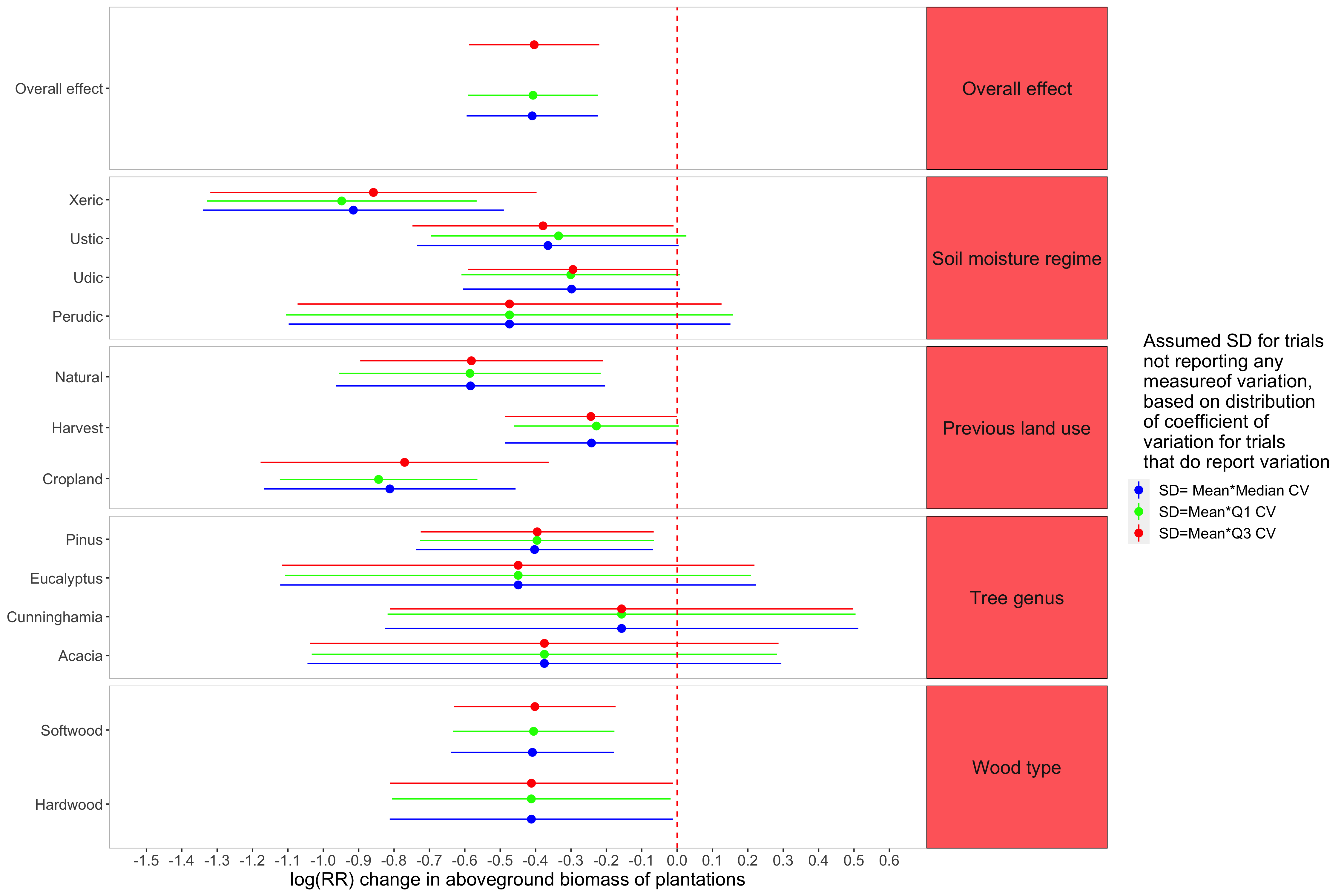


**Figure S4.3:** Sensitivity analysis of thinning meta-analysis to imputing missing standard d

*S5| Regression result tables*

**Table S5.1**: The regression results of the effect of each continuous variable, and of each category of categorical moderator variables on the magnitude of the effect size of interplanting of N-fixing plants. * indicates p = 0.01–0.05; ** indicates p = 0.001–0.01; *** indicates p < 0.001.

|  |  | No. Obs. | Estimate | Std. error | z value | Pr(>\|z\|) | Significance |
| --- | --- | --- | --- | --- | --- | --- | --- |
| Overall effect | Cumulated effect | 197 | -0.0154 | 0.077 | -0.2005 | 0.8411 |  |
| Continuous moderator variables | (Intercept) |  | -0.0955 | 0.0556 | -1.7181 | 0.0858 |  |
|  | Stand age | 195 | 0.0355 | 0.0017 | 20.4051 | <.0001 | *** |
|  | (Intercept) |  | 0.0515 | 0.1006 | 0.5118 | 0.6088 |  |
|  | Precipitation | 197 | 0.0001 | 0.0001 | 1.5230 | 0.1278 |  |
| Soil moisture regime | Perudic | 40 | 0.4234 | 0.1642. | 2.5790 | 0.0099 | ** |
|  | Udic | 139 | 0.1296 | 0.0677 | 1.9149 | 0.0555 |  |
|  | Ustic | 12 | 0.3550 | 0.0857 | 4.1439 | <.0001 | *** |
|  | Xeric | 66 | 0.0222 | 0.2734 | 0.0813 | 0.9352 |  |
| Previous land use | Cropland | 69 | 0.5111 | 0.1272 | 4.0193 | <.0001 | *** |
|  | Plantation | 41 | 0.2149 | 0.1366 | 1.5730 | 0.1157 |  |
|  | Natural forest | 21 | -0.1918 | 0.1387 | -1.3830 | 0.1667 |  |
| Tree Genus | Eucalyptus | 142 | 0.2322 | 0.0852 | 2.7263 | 0.0064 | ** |
|  | Alnus | 2 | -0.4079 | 0.3972 | -1.0269 | 0.3044 |  |
|  | Anacardium | 2 | -0.3537 | 0.3949 | -0.8955 | 0.3705 |  |
|  | Pachira | 2 | 0.6704 | 0.3949 | 1.6975 | 0.0896 |  |
|  | Casuarina | 3 | -0.0735 | 0.1366 | -0.5376 | 0.5909 |  |
|  | Pinus | 20 | 0.2170 | 0.1426 | 1.5219 | 0.1280 |  |
|  | Populus | 15 | 0.0847 | 0.1838 | 0.4606 | 0.6451 |  |
|  | Pseudotsuga | 4 | -0.0682 | 0.4007 | -0.1702 | 0.8648 |  |
| Intercropped plant genus | Acacia | 71 | 0.1045 | 0.0866 | 1.2062 | 0.2277 |  |
|  | Albizia | 25 | 0.5931 | 0.2021 | 2.9343 | 0.0033 | ** |
|  | Alnus | 15 | -0.0855 | 0.1452 | -0.5891 | 0.5558 |  |
|  | Betula | 5 | -0.1885 | 0.1600 | -1.1783 | 0.2387 |  |
|  | Callophylum |  | 0.0558 | 0.5408 | 0.1031 | 0.9179 |  |
|  | Casuarina | 6 | 0.0268 | 0.1310 | 0.2043 | 0.8381 |  |
|  | Dalbergia | 4 | 0.1584 | 0.3395 | 0.4665 | 0.6409 |  |
|  | Enterolobium | 4 | 0.6285 | 0.2035 | 3.0877 | 0.0020 | ** |
|  | Falcataria | 25 | 0.4070 | 0.3390 | 3.0088 | 0.0026 |  |
|  | Hippophae | 3 | 0.7592 | 0.2523 | 1.2004 | 0.2300 | ** |
|  | Leucaena | 7 | 0.4191 | 0.1975 | 2.1217 | 0.0339 | ** |
|  | Lupinus | 4 | 0.3259 | 0.3415 | 0.9543 | 0.3399 |  |
|  | Ormosia |  | 0.4338 | 0.3422 | 1.2675 | 0.2050 |  |
|  | Paraserianthes | 4 | 0.3016 | 0.2035 | 1.5257 | 0.1271 |  |
|  | Robinia | 19 | 0.1719 | 0.1597 | 1.0764 | 0.2817 |  |
|  | Salix | 2 | -0.4078 | 0.3428 | -1.1899 | 0.2341 |  |
| Wood type | Hardwood | 167 | 0.2231 | 0.0674 | 3.3078 | 0.0009 | *** |
|  | Softwood | 30 | 0.0597 | 0.0906 | 0.6586 | 0.5101 |  |
| Experimental design | Additive  Replacement | 166  31 | 0.5472  0.1503 | 0.0761  0.0694 | 7.1950  2.1648 | <.0001  0.0304 | ***  * |

**Table S5.2**: The regression results of the effect of each continuous variable, and of each category of categorical moderator variables on the magnitude of the effect size of NPK fertilization. * indicates p = 0.01–0.05; ** indicates p = 0.001–0.01; *** indicates p < 0.001.

|  |  | No. Obs. | Estimate | Std. error | z value | Pr(>\|z\|) | Significance |
| --- | --- | --- | --- | --- | --- | --- | --- |
|  | Overall effect | 164 | 0.3686 | 0.0683 | 5.3999 | <.0001 | *** |
| Continuous moderator variables | (Intercept) |  | 0.3530 | 0.0889 | 3.9697 | <.0001 | *** |
|  | Stand age | 112 | -0.0039 | 0.0059 | -0.6650 | 0.5051 |  |
|  | (Intercept) |  | 0.6144 | 0.1270 | 4.8395 | <0.001 | *** |
|  | Time since fertilization | 49 | -0.0689 | 0.0258 | -2.6692 | 0.0076 | ** |
|  | (Intercept) |  | 0.7536 | 0.135 | 5.5833 | <.0001 | *** |
|  | Precipitation | 164 | -0.0003 | 0.0001 | -3.5709 | 0.0004 | *** |
| Soil moisture regime | Perudic | 4 | 0.6084 | 0.2893 | 2.1033 | 0.0354 | * |
|  | Udic | 155 | 0.3866 | 0.0754 | 5.1247 | <.0001 | *** |
|  | Ustic | 3 | -0.0122 | 0.291 | -0.042 | 0.9664 |  |
|  | Xeric | 2 | 0.2336 | 0.2953 | 0.7912 | 0.4288 |  |
| Previous land use | Cropland | 28 | 0.22 | 0.2048 | 1.0839 | 0.2784 |  |
|  | Plantation | 96 | 0.3659 | 0.1326 | 2.7593 | 0.0058 | ** |
|  | Fire | 4 | 0.273 | 0.2866 | 0.9528 | 0.3407 |  |
|  | Natural forest | 15 | 0.565 | 0.1298 | 4.3526 | <.0001 | *** |
| Tree genus | Ailanthus | 3 | -0.0122 | 0.2049 | -0.0598 | 0.9523 |  |
|  | Eucalyptus | 110 | 0.3183 | 0.0947 | 3.3611 | 0.0008 | *** |
|  | Macaranga | 4 | 0.3308 | 0.2024 | 1.6344 | 0.1022 |  |
|  | Picea | 3 | 0.0806 | 0.1477 | 0.5456 | 0.5854 |  |
|  | Pinus | 42 | 0.4167 | 0.0716 | 5.8156 | <.0001 | *** |
| Application method | Continuous | 140 | 0.3080 | 0.0801 | 3.8468 | 0.0001 | *** |
|  | Pulse | 24 | 0.4280 | 0.0789 | 5.3657 | <.0001 | *** |
| Wood type | Hardwood | 120 | 0.3812 | 0.1063 | 3.5863 | 0.0003 | *** |
|  | Softwood | 44 | 0.3584 | 0.0936 | 3.8272 | 0.0001 | *** |

**Table S5.3**: The regression results of the effect of each continuous variable, and of each category of categorical moderator variables on the magnitude of the effect size of thinning. * indicates p = 0.01–0.05; ** indicates p = 0.001–0.01; *** indicates p < 0.001.

|  |  | No. Obs. | Estimate | Std. error | z value | Pr(>\|z\|) | Significance |
| --- | --- | --- | --- | --- | --- | --- | --- |
|  | Overall effect | 62 | -0.4095 | 0.0946 | -4.271 | <.0001 | *** |
| Continuous moderator variables | (Intercept) |  | -0.6482 | 0.0802 | -8.0797 | <.0001 | *** |
|  | Time since thinning | 62 | 0.0443 | 0.005 | 8.8869 | <.0001 | *** |
|  | (Intercept) |  | -0.8071 | 0.2215 | -3.6445 | 0.0003 | *** |
|  | Precipitation | 62 | 0.0003 | 0.0001 | 2.0298 | 0.0424 | * |
|  | (Intercept) |  | 0.3469 | 0.0906 | 3.8293 | 0.0001 | *** |
|  | Basal area removed | 57 | -0.0140 | 0.0013 | -10.8587 | <.0001 | *** |
| Soil moisture regime | Perudic | 2 | -0.4736 | 0.3186 | -1.4866 | 0.1371 |  |
|  | Udic | 11 | -0.2982 | 0.1567 | 1.9036 | 0.057 |  |
|  | Ustic | 31 | -0.365 | 0.1885 | -1.9366 | 0.0528 |  |
|  | Xeric | 18 | -0.9151 | 0.217 | -4.2172 | <.0001 | *** |
| Previous land use | Cropland | 17 | -0.8119 | 0.1813 | -4.479 | <.0001 | *** |
|  | Plantation | 36 | -0.242 | 0.1246 | -1.9417 | 0.0522 |  |
|  | Natural forest | 5 | -0.5838 | 0.1941 | -3.0079 | 0.0026 | ** |
| Tree Genus | Acacia | 3 | -0.3751 | 0.3417 | -1.0978 | 0.2723 |  |
|  | Cunninghamia | 2 | -0.1569 | 0.3415 | -0.4595 | 0.6459 |  |
|  | Eucalyptus | 4 | -0.4492 | 0.3432 | -1.3091 | 0.1905 |  |
|  | Pinus | 50 | -0.4029 | 0.171 | -2.3567 | 0.0184 | * |
| Wood type | Hardwood | 7 | -0.4119 | 0.2043 | -2.0164 | 0.0438 | * |
|  | Softwood | 55 | -0.4089 | 0.1178 | -3.4723 | 0.0005 | *** |

*S6| Studies used for the meta-analysis (studies selected from Bukoski et al. (2022) database and Feng et al. (2022))*

1. Austin MT, Brewbaker JL, Wheeler R, Fownes JH (1997) Short-rotation biomass trial of mixed and pure stands of nitrogen-fixing trees and Eucalyptus grandis. Australian Forestry 60:161–168

2. Balieiro FC, Fontes RLF, Dias LE, Franco AA, Campello EFC, de Faria SM (2002) Accumulation and Distribution of Aboveground Biomass and Nutrients in Pure and Mixed Stands of Guachapele and Eucalyptus. Journal of Plant Nutrition 25:2639–2654

3. Beets P, Whitehead D (1996) Carbon partitioning in Pinus radiata stands in relation to foliage nitrogen status. Tree physiology 16:131–138

4. Bennett LT, Weston CJ, Attiwill PM (1997) Biomass, Nutrient Content and Growth Response to Fertilisers of Six-year-old Eucalyptus globulus Plantations at Three Contrasting Sites in Gippsland, Victoria. Aust J Bot 45:103–121

5. Binkley D, Senock R, Bird S, Cole TG (2003) Twenty years of stand development in pure and mixed stands of Eucalyptus saligna and nitrogen-fixing Facaltaria moluccana. Forest Ecology and Management 182:93–102

6. Bogdan S, Sporcic M, Seletković Z, Ivanković M (2009) Biomass Production of Common Alder (Alnus glutinosa /L./ Gaertn.) in Pure Plantations and Mixed Plantations with Willow Clones (Salix sp.) in Croatia (p.99-112). Croatian Journal of Forest Engineering

7. Bouillet J-P, Laclau J-P, Gonçalves JL de M, et al (2013) Eucalyptus and Acacia tree growth over entire rotation in single- and mixed-species plantations across five sites in Brazil and Congo. Forest Ecology and Management 301:89–101

8. Chang SX, Preston CM (2000) Understorey competition affects tree growth and fate of fertilizer-applied 15N in a Coastal British Columbia plantation forest: 6-year results. Can J For Res 30:1379–1388

9. Chen X (2009) Stand biomass and its distribution of mixed forests of Alnus cremastogyne and Cunninghamia lanceolata. South China Forestry Science. South China Forestry Science 14–17

10. Cheng X, Yu M, Wang GG (2017) Effects of Thinning on Soil Organic Carbon Fractions and Soil Properties in Cunninghamia lanceolata Stands in Eastern China. Forests 8:198

11. Debell DS, Cole TG, Whitesell CD (1997) Growth, Development, and Yield in Pure and Mixed Stands of Eucalyptus and Albizia. Forest Science 43:286–298

12. Epron D, Nouvellon Y, Mareschal L, et al (2013) Partitioning of net primary production in Eucalyptus and Acacia stands and in mixed-species plantations: Two case-studies in contrasting tropical environments. Forest Ecology and Management. https://doi.org/10.1016/j.foreco.2012.10.034

13. Forrester D, Bauhus J, Khanna P (2004) Growth dynamics in a mixed-species plantation of Eucalyptus globulus and Acacia mearnsii. Forest Ecology and Management 193:81–95

14. Forrester DI, Collopy JJ, Beadle CL, Baker TG (2012) Interactive effects of simultaneously applied thinning, pruning and fertiliser application treatments on growth, biomass production and crown architecture in a young Eucalyptus nitens plantation. Forest Ecology and Management 267:104–116

15. Forrester DI, Schortemeyer M, Stock WD, Bauhus J, Khanna PK, Cowie AL (2007) Assessing nitrogen fixation in mixed- and single-species plantations of Eucalyptus globulus and Acacia mearnsii. Tree Physiology 27:1319–1328

16. Forrester D, Theiveyanathan S, Collopy J, Marcar N (2010) Enhanced water use efficiency in a mixed Eucalyptus globulus and Acacia mearnsii plantation. Forest Ecology and Management 259:1761–1770

17. Fredericksen TS (1991) Interference interactions in experimental pine-hardwood stands.

18. Gholz H, VOGEL S, Cropper W, McKelvey K, Ewel K, Teskey R, Curran P (1991) Dynamics of Canopy Structure and Light Interception in Pinus Elliottii Stands, North Florida. ECOLOGICAL MONOGRAPHS 61:33–51

19. Ghorbani M, Sohrabi H, Sadati E, Babaei F (2018) Productivity and dynamics of pure and mixed-species plantations of Populous deltoids Bartr. ex Marsh and Alnus subcordata C. A. Mey. Forest Ecology and Management 409:890–898

20. Gresham CA (2002) Sustainability of intensive loblolly pine plantation management in the South Carolina Coastal Plain, USA. Forest Ecology and Management 155:69–80

21. Groninger JW, Zedaker SM, Fredericksen TS (1997) Stand characteristics of inter-cropped loblolly pine and black locust. Forest Ecology and Management 91:221–227

22. Han J, Yao Q, Hong C, Lin W (2008) Study on the growth and ecological benefit of mixed stand of Eucalyptus grandis x urophylla and Acacia mangium x auriculiformis. Eucalypt Science and Technology 25:15–18

23. Hennessey TC, Dougherty PM, Lynch TB, Wittwer RF, Lorenzi EM (2004) Long-term growth and ecophysiological responses of a southeastern Oklahoma loblolly pine plantation to early rotation thinning. Forest Ecology and Management 192:97–116

24. Ingerslev M, Hallbäcken L (1999) Above ground biomass and nutrient distribution in a limed and fertilized Norway spruce (Picea abies) plantation: Part II. Accumulation of biomass and nutrients. Forest Ecology and Management 119:21–38

25. Jaquetti RK, Gonçalves JFC (2017) Carbon and nutrient stocks of three Fabaceae trees used for forest restoration and subjected to fertilization in Amazonia. An Acad Bras Cienc 89:1761–1771

26. Jokela E, Martin T (2000) Effects of ontogeny and soil nutrient supply on production, allocation, and leaf area efficiency in loblolly and slash pine stands. Canadian Journal of Forest Research-Revue Canadienne de Recherche Forestiere 30:1511–1524

27. Kaye JP, Resh SC, Kaye MW, Chimner RA (2000) Nutrient and Carbon Dynamics in a Replacement Series of Eucalyptus and Albizia Trees. Ecology 81:3267–3273

28. Kim C, Son Y, Lee W-K, Jeong J, Noh NJ (2013) Influences of forest tending works on carbon distribution and cycling in a Pinus densiflora S. et Z. stand in Korea. Forest Ecology and Management 257:1420–1426

29. Kunhamu TK, Kumar BM, Viswanath S (2009) Does thinning affect litterfall, litter decomposition, and associated nutrient release in Acacia mangium stands of Kerala in peninsular India? Can J For Res 39:792–801

30. Laclau J-P, Bouillet J-P, Gonçalves JLM, et al (2008) Mixed-species plantations of Acacia mangium and Eucalyptus grandis in Brazil: 1. Growth dynamics and aboveground net primary production. Forest Ecology and Management 255:3905–3917

31. le Maire G, Nouvellon Y, Christina M, Ponzoni F, Gonçalves J, Jean-Pierre B, Laclau J-P (2013) Tree and stand light use efficiencies over a full rotation of single- and mixed-species Eucalyptus grandis and Acacia mangium plantations. Forest Ecology and Management 288:31–42

32. Li B, Tang G, Li K, Gao C, Liu F, Wang X (2013) Vegetation biomass allocation and its spatial distribution after 20 years ecological restoration in a dry-hot valley in Yuanmou, Yunnan Province of Southwest China. Chinese Journal of Applied Ecology. Chinese Journal of Applied Ecology 24:1479–1486

33. Liu X, Lu Y, Xie Y, Xue Y (2015) The Positive Interaction Between Two Nonindigenous Species, Casuarina (Casuarina Equisetifolia) and Acacia (Acacia Mangium), in the Tropical Coastal Zone of South China: Stand Dynamics and Soil Nutrients. Tropical Conservation Science 8:598–609

34. Liu Y (2017) Preliminary report on the effect of mixed afforestation of Cunninghamia lanceolata and Betula luminifera. Journal of Green Science and Technology. https://doi.org/10.16663/j.cnki.lskj.2017.13.007

35. Madeira M, Fabião A, Pereira J, Araujo M, Ribeiro C (2002) Changes in carbon stocks in Eucalyptus globulus Labill. plantations induced by different water and nutrient availability. Forest Ecology and Management 171:75–85

36. Maier CA, Albaugh TJ, Lee Allen H, Dougherty PM (2004) Respiratory carbon use and carbon storage in mid-rotation loblolly pine ( *Pinus taeda* L.) plantations: the effect of site resources on the stand carbon balance: STAND RESPIRATION AND CARBON STORAGE. Global Change Biology 10:1335–1350

37. Mao R, Zeng D-H, Ai G-Y, Yang D, Li L-J, Liu Y-X (2010) Soil microbiological and chemical effects of a nitrogen-fixing shrub in poplar plantations in semi-arid region of Northeast China. European Journal of Soil Biology 46:325–329

38. Marron N, Priault P, Gana C, Gérant D, Epron D (2018) Prevalence of interspecific competition in a mixed poplar/black locust plantation under adverse climate conditions. Annals of Forest Science 75:1–12

39. Mayoral C, van Breugel M, Cerezo A, Hall JS (2017) Survival and growth of five Neotropical timber species in monocultures and mixtures. Forest Ecology and Management 403:1–11

40. Moore G, Bond B, Jones J (2011) A comparison of annual transpiration and productivity in monoculture and mixed-species Douglas-fir and red alder stands. Forest Ecology and Management 262:2263–2270

41. Oliveira N, del Río M, Forrester DI, Rodríguez-Soalleiro R, Pérez-Cruzado C, Cañellas I, Sixto H (2018) Mixed short rotation plantations of Populus alba and Robinia pseudoacacia for biomass yield. Forest Ecology and Management 410:48–55

42. Pan C, Yang F, Lan P, Li Y (1998) Characteristics of soil microbes in south subtropical lateritic red earth under artificial forests. Journal of Tropical and Subtropical Botany 6:158–165

43. Parrotta J (1999) Productivity, nutrient cycling, and succession in single- and mixed-species plantations of Casuarina equisetifolia, Eucalyptus robusta, and Leucaena leucocephala in Puerto Rico. Forest Ecology and Management - FOREST ECOL MANAGE 124:45–77

44. Redondo-Brenes A, Montagnini F (2006) Growth, productivity, aboveground biomass, and carbon sequestration of pure and mixed native tree plantations in the Caribbean lowlands of Costa Rica. Forest Ecology and Management 232:168–178

45. Resh SC, Battaglia M, Worledge D, Ladiges S (2003) Coarse root biomass for eucalypt plantations in Tasmania, Australia: sources of variation and methods for assessment. Trees 17:389–399

46. Ruiz-Mirazo J, Gonzalez-Rebollar JL (2013) Growth and structure of a young Aleppo pine planted forest after thinning for diversification and wildfire prevention. Forest Systems 22:47–57

47. Ryan MG, Binkley D, Fownes JH, Giardina C, Senock RS (2004) An experimental test of the causes of forest growth decline with stand age. Ecological Monograph, 74(3), 2004, pp. 393-414.

48. Samuelson LJ, Johnsen K, Stokes T (2004) Production, allocation, and stemwood growth efficiency of Pinus taeda L. stands in response to 6 years of intensive management. Forest Ecology and Management 192 (2004) 59-­70

49. Santos F, Balieiro F, Ataíde D, Diniz A, Chaer G (2016) Dynamics of aboveground biomass accumulation in monospecific and mixed-species plantations of Eucalyptus and Acacia on a Brazilian sandy soil. Forest Ecology and Management 363:86–97

50. Schweier J, Arranz C, Nock C, Jaeger D, Scherer-Lorenzen M (2019) Impact of Increased Genotype or Species Diversity in Short Rotation Coppice on Biomass Production and Wood Characteristics. BioEnergy Research. https://doi.org/10.1007/s12155-019-09997-2

51. Shan J, Morris LA, Hendrick RL (2001) The effects of management on soil and plant carbon sequestration in slash pine plantations. Journal of Applied Ecology 38:932–941

52. Shujauddin N, Kumar BM (2003) Ailanthus triphysa at different densities and fertiliser regimes in Kerala, India: growth, yield, nutrient use efficiency and nutrient export through harvest. Forest Ecology and Management 180:135–151

53. Sicard C, Saint-Andre L, Gelhaye D, Ranger J (2006) Effect of initial fertilisation on biomass and nutrient content of Norway Spruce and Douglas-Fir plantations at the same site. Trees 20:229–246

54. Son Y, Lee YY, Lee CY, Yi MJ (2007) Nitrogen Fixation, Soil Nitrogen Availability, and Biomass in Pure and Mixed Plantations of Alder and Pine in Central Korea. Journal of Plant Nutrition 30:1841–1853

55. Stape JL, Binkley D, Ryan MG, et al (2010) The Brazil Eucalyptus Potential Productivity Project: Influence of water, nutrients and stand uniformity on wood production. Forest Ecology and Management 259:1684–1694

56. Subedi P, Jokela EJ, Vogel JG, Martin TA Inter-rotational Effects of Fertilization and Weed Control on Juvenile Loblolly Pine Productivity and Nutrient Dynamics.

57. Susanto D (2017) Growth, biomass production and nutrient accumulation of Macaranga gigantea in response to NPK fertilizer application.

58. Toïgo M, Castagneyrol B, Jactel H, Morin X, Meredieu C (2022) Effects of tree mixture on forest productivity: tree species addition versus substitution. European Journal of Forest Research. https://doi.org/10.1007/s10342-021-01432-6

59. Vogel J, He D, Jokela E, Hockaday W, Schuur E (2015) The effect of fertilization levels and genetic deployment on the isotopic signature, constituents, and chemistry of soil organic carbon in managed loblolly pine (Pinus taeda L.) forests. Forest Ecology and Management. https://doi.org/10.1016/j.foreco.2015.05.020

60. Weng J (2008) Study on the effect of different mixed ratio afforestation methods of Ormosia hosiei and Cunninghamia lanceolata. Anhui Agricultural Science Bulletin 14:157–159

61. Xiao W, Wang S, Chen X, Xiao L, Wu G, Luo Z (1999) Economic benefits analysis of Eucalyptus and Acacia crassicarpa mixed forests. Forestry and Environmental Science 2:2–8

62. Xu J (1998) Studies on planting effects of the mixed forest of Pinus elliottii and Casuarina equisetifolia on coastal belts. Journal of Fujian Forestry Science and Technology 25:28–32

63. Yang H (2015) Management technique of artifical mixed plantation of Eucalyptus and Acacia concinnatai. Forest Inventory and Planning 40:91–95

64. Yang Z-J, Xu D-P, Chen W-P, Huang L-J, Li S-J, Chen Y (2009) Growth effect of eucalyptus-acacia mixed plantation in South China. Ying Yong Sheng Tai Xue Bao 20:2339–2344

65. Ye FG (2002) Studies on the Establishment and Control Techniques of Mixed Forest of Casuarina equisetifolia and Pinus elliottii in Coast Zone. Forest Research 15:463–468

66. Ye M (2013) Effect analysis of Pinus elliottii mixed with Acacia mangium. South China Forestry Science 4:11–12

67. Zhang J-T, Chen T (2007) Effects of mixed Hippophae rhamnoides on community and soil in planted forests in the Eastern Loess Plateau, China. Ecological Engineering 31:115–121

68. Zou L (2006) Study on productivity and soil physical and chemical property of mixed stand of Pinus massoniana with Acacia dealbata Link. Protection Forest Science and Technology, 74:5–7

*S7| References for the “S2 | Description of soil moisture regimes” section*

1. USDA. Illustrated Guide to Soil Taxonomy, version 2.0 [Internet]. Lincoln: US Department of Agriculture; 2015. Available from: https://www.nrcs.usda.gov/wps/portal/nrcs/detail/soils/survey/class/taxonomy/?cid=nrcs142p2_053580

2. Buol S. Tropical Soils | Humid Tropical. Reference Module in Earth Systems and Environmental Sciences. 2013.

3. Chesworth W, editor. Encyclopedia of Soil Science [Internet]. Dordrecht: Springer Netherlands; 2008 [cited 2022 Mar 14]. Available from: http://link.springer.com/10.1007/978-1-4020-3995-9

4. Rossiter. Description of Soil Moisture Regimes according to USDA Soil Taxonomy 8th edition of Keys [Internet]. 2020 [cited 2022 Mar 14]. Available from: http://www.css.cornell.edu/faculty/dgr2/_static/legacy_sw/nsm/nsm_SMR.html
